# Mass Drug Administration to Reduce Malaria Transmission: A Systematic Review and Meta-Analysis

**DOI:** 10.4269/ajtmh.22-0766

**Published:** 2023-12-20

**Authors:** Zachary D. Schneider, Monica P. Shah, Marisa C. Boily, Alexandra L. Busbee, Jimee Hwang, Kim A. Lindblade, Julie R. Gutman

**Affiliations:** ^1^ Malaria Branch, Division of Parasitic Diseases and Malaria, Centers for Disease Control and Prevention, Atlanta, Georgia;; ^2^ Rollins School of Public Health, Emory University, Atlanta, Georgia;; ^3^ U.S. President’s Malaria Initiative, Malaria Branch, Division of Parasitic Diseases and Malaria, Centers for Disease Control and Prevention, Atlanta, Georgia;; ^4^ Global Malaria Programme, World Health Organization, Geneva, Switzerland

## Abstract

Malaria remains a significant cause of morbidity and mortality, even in low-transmission settings. With the advent of longer acting, more effective, and well-tolerated antimalarials, there is renewed interest in the efficacy of mass drug administration (MDA) to accelerate to elimination. We conducted a systematic review and meta-analysis to assess the efficacy of MDA to reduce the incidence and prevalence of *Plasmodium falciparum* (*Pf*) and *Plasmodium vivax* (*Pv*) infection. From 1,044 articles screened, 14 articles, including 10 randomized controlled trials (RCTs), were identified. Five included data on *Pf* only; five included *Pf* and *Pv*. Two of the *Pf* studies were conducted in areas of high–moderate transmission, the remainder were in areas of low–very low transmission. In higher transmission areas, MDA reduced incidence of *Pf* parasitemia (rate ratio = 0.61, 95% CI: 0.40–0.92; moderate certainty) 1 to 3 months after drug administration; no significant effect of MDA on *Pf* parasitemia prevalence was detected 1 to 3 months post-MDA (risk ratio [RR] = 1.76, 95% CI: 0.58–5.36; low certainty). In lower transmission settings, both incidence and prevalence of *Pf* parasitemia were reduced 1 to 3 months post-MDA (rate ratio = 0.37, 95% CI: 0.21–0.66; RR = 0.25, 95% CI: 0.15–0.41, respectively). *Pv* prevalence was reduced 1 to 3 months post-MDA (RR = 0.15, 95% CI: 0.10–0.24); there were no RCTs providing data on incidence of *Pv*. There was no significant effect of MDA at later time points. MDA may have short-term benefits; however, there was no evidence for longer term impact, although none of the trials assessed prolonged interventions.

## INTRODUCTION

Mass drug administration (MDA) is the administration of a full course of antimalarial medication (irrespective of the presence of symptoms or infection) to every person living in a defined geographic area (except to those for whom the medicine is contraindicated) at approximately the same time and often at repeated intervals.^1^ Mass drug administration targets the human parasite reservoir by both clearing existing parasites from the population and providing a variable prophylactic window during which new infections are prevented. The effectiveness of MDA depends on the duration and effectiveness of the selected antimalarial, co-deployment of vector control, the timing of rounds in relation to seasonal malaria peaks, and, most importantly, MDA coverage, which can be improved by ensuring a high proportion of the population participates in each round as well as providing multiple rounds (thus covering a greater proportion of the population).^2^^–^^4^

Mass drug administration has the potential to reduce community-level transmission through reduction in the human reservoir of infection and prevention of future infections. Older studies have found large reductions in parasite prevalence immediately after MDA rounds; however, these gains generally were not sustained.^5^ With the availability of longer acting, well-tolerated antimalarials (e.g., artemisinin combination therapies; ACTs) and drugs with gametocidal effects, there is renewed interest in MDA as an intervention for malaria elimination.^6^ Mathematical modeling studies have suggested that reductions in transmission are temporary with greater impact predicted for lower transmission settings, with higher coverage (80–90%) and three rounds per year continued over 2 years.^3^^,^^7^ Mass drug administration has contributed to successful interruption of malaria transmission in only a few instances, mainly in geographically isolated settings and always in conjunction with additional interventions. Notable examples include malaria elimination on the island of Aneityum, Vanuatu^8^ where MDA was combined with larvivorous fish and vector control, and on the island of Lanyu, Taiwan,^9^^,^^10^ in combination with indoor residual spraying (IRS). In addition, MDA was used in China as part of a successful, multifaceted elimination program.^11^^,^^12^

Mass drug administration approaches specific for *Plasmodium vivax* and *Plasmodium ovale* may require additional measures to address the latent, dormant stage in the liver, the hypnozoites. Hypnozoites can cause relapses months, or even years, after initial infection and a MDA strategy targeting these parasites may include the addition of a drug to reduce the risk and rate of future relapses. Currently, only 8-aminoquinolines are effective against the liver stage of these parasites. Because 8-aminoquinolines can cause severe hemolysis among persons with a deficiency of glucose-6-phosphate dehydrogenase, an enzyme that helps red blood cells function, additional testing and pharmacovigilance are required when treating people with these drugs. Additionally, the drug currently recommended by the WHO to treat hypnozoites, primaquine, requires a multiday regimen. These considerations mean that recommendations related to MDA for *Plasmodium vivax* (or *P. ovale*, a parasite found much less frequently) are likely to be quite different from those for *P. falciparum*.

A WHO advisory group reviewed evidence on MDA in 2015 and recommended that MDA be considered for the elimination of *P. falciparum* in areas approaching interruption of transmission and where other interventions were already in place.^13^ Mass drug administration was additionally recommended to reduce transmission of *P. falciparum* in the Greater Mekong subregion, where there was a looming threat from increased parasite resistance to antimalarials, and for epidemic control or in complex emergencies. Mass drug administration was not recommended for *P. vivax.* An updated review of MDA was conducted in 2018 to incorporate new trials, but no official recommendations were developed because the WHO was in the midst of updating its process for developing malaria guidelines.^14^ In 2021, the WHO commissioned this systematic review and meta-analysis of the impact of MDA on the transmission of *P. falciparum* and *P. vivax* to inform a new guideline development.

## MATERIALS AND METHODS

The methods for this systematic review have been described extensively elsewhere in this supplement^15^ and in the prospectively published protocol [PROSPERO registration: CRD42021240921]. Specific attributes of the methods for this review are noted in this section.

### Population, intervention, comparison, and outcomes.

The population included adults and children living in delimited geographic areas with ongoing malaria transmission. Studies in special groups (i.e., refugees and soldiers) were included if they met eligibility criteria and the intervention was administered to the entire population of a defined geographic area.

The intervention was defined as the administration of a full therapeutic course of antimalarial medicine (irrespective of the presence of symptoms or infection) to every person living in a defined geographic area (except for those for whom the medicine is contraindicated) at approximately the same time (synchronous) and often at repeated intervals. The comparison was no intervention or placebo.

For the effect on *P. falciparum*, antimalarials for MDA included drugs that act on blood-stage parasites alone or in combination with a gametocidal drug, but there were no restrictions on the specific antimalarials considered. We included only studies that provided doses of antimalarials intended for curative purposes. Any dose greater than the current standard prophylactic dose (if applicable) was considered as a therapeutic dose (e.g., chloroquine or amodiaquine at 300 mg base weekly, pyrimethamine at 25 mg weekly, proguanil at 100 mg daily, mepacrine at 300 mg weekly in one dose or 700 mg weekly in daily doses of 100 mg, and quinine at 325 mg twice a day).^16^^,^^17^ The minimum dosage of primaquine to be considered effective as a gametocide was a single dose of 0.25 mg/kg.

For the effect of MDA on *P. vivax*, the drug regimen included drugs for blood stages alone or both blood and liver stages (mass relapse prevention, or the mass administration of drugs for liver stages alone, is the subject of another review in this supplement^18^). The therapeutic dose of primaquine to treat hypnozoites was a minimum of 3.5 mg/kg total dose administered over up to 8 weeks.

Critical outcomes including incidence of parasitemia and parasite prevalence were measured at the population-level as previously described.^15^ Incidence of clinical malaria, a secondary outcome, included confirmed malaria identified through passive surveillance as a result of symptomatic cases seeking care or active case detection where symptoms were noted in addition to parasitemia. Certain outcomes (i.e., adverse events and drug resistance markers) were measured only among those receiving the intervention. If outcomes were reported for unspecified *Plasmodium* species, the local epidemiology of the study area was used to infer the predominant species. Data were pooled into the following post-MDA time periods: <1 month, 1–3 months, 4 to <12 months and 12-24 months following the last round of MDA.

### Data collection and analysis.

The selection of studies, data extraction, assessment of risk of bias, and data synthesis have been described previously.^15^ Because a Cochrane review of MDA had been completed in 2013,^5^ all studies identified in that systematic review were screened for inclusion for this review. For the period after the review was completed (i.e., from February 2013), the search terms and search strategy were similar to the Cochrane Review and are presented in Supplemental Table 1.

The following factors were abstracted to determine whether the impact of MDA differed by potential effect modifiers: transmission level (very low to low, moderate to high),^19^ medication used for blood stage clearance as well as for radical cure (primaquine or tafenoquine), coverage of the intervention (≥80% versus <80%), timing of the intervention with respect to the transmission season, duration of implementation, geographic isolation of the study area, and coverage of insecticide treated nets and indoor residual spraying. The certainty of evidence for each outcome was assessed by the Grading of Recommendations, Assessment, Development and Evaluations process.^20^

## RESULTS

**Figure 1.**
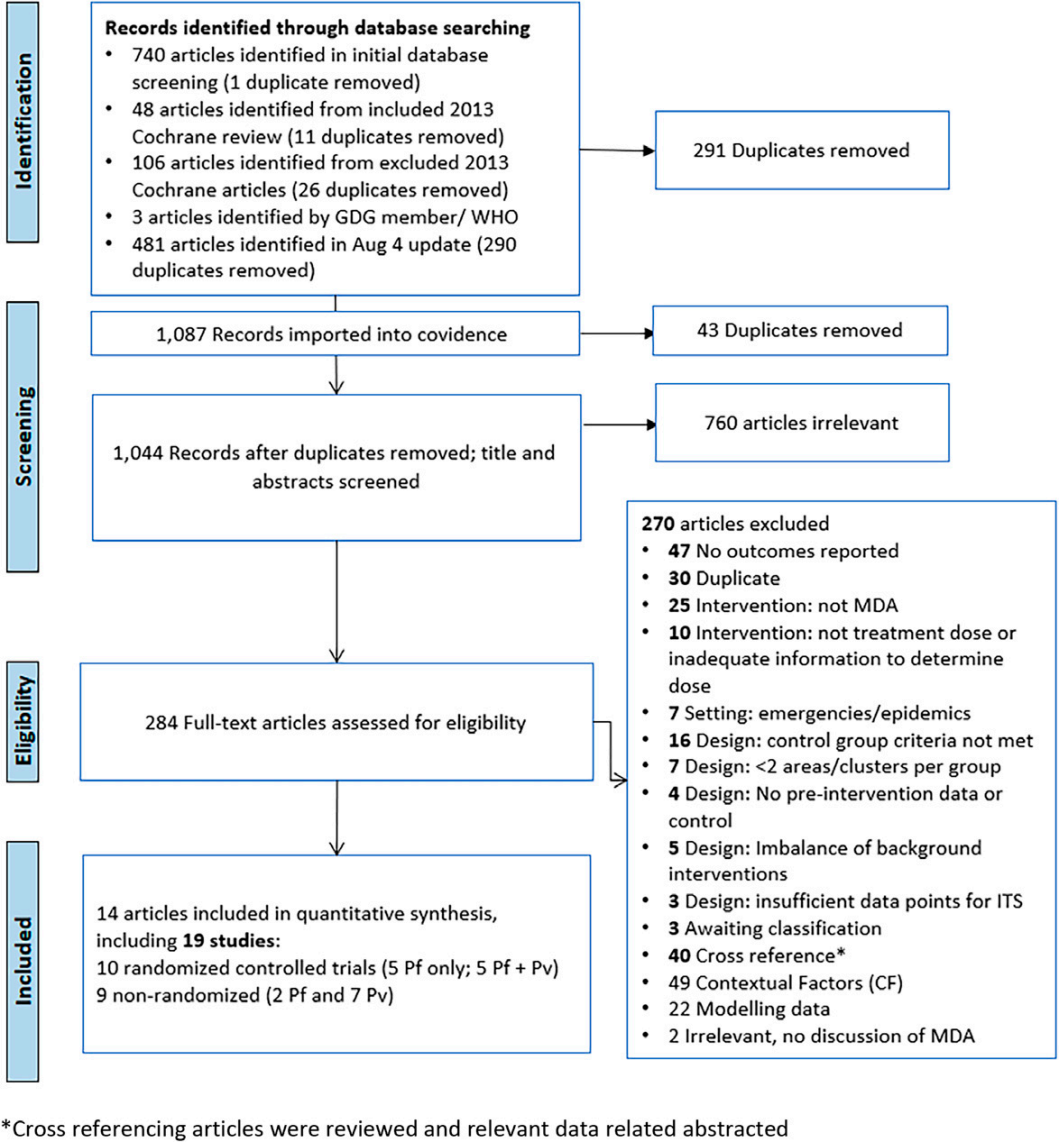
PRISMA flow diagram. Pf = *P. falciparum*, Pv = *P. vivax*, WHO = World Health Origination.

A total of 1,367 articles was identified from searching electronic databases, registers, and other sources: 1,221 records from database search from 2012 onward (date of search: November 11, 2020; updated August 4, 2022; Supplemental Table 1), 143 from a previous Cochrane Review on MDA,^5^ and an additional three from other sources. After de-duplication, 1,044 articles were screened against title and abstract for potential eligibility, and of these, 284 full texts were assessed for eligibility (Figure 1). Full-text studies that did not meet eligibility criteria (see Methodology paper) are listed with the reasons for exclusion in Supplemental Table 2.

**Figure 2.**
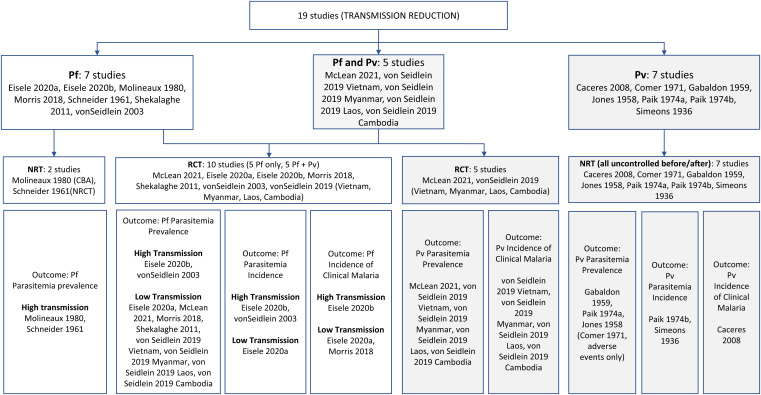
Classification of included studies.

A total of 14 articles reporting on 19 studies were included in the review (Figure 2; 40 articles designated as cross-references with relevant additional data were also reviewed and abstracted): seven studies provided data on *P. falciparum* alone—five cluster-randomized controlled studies (cRCTs)^21^^–^^24^ and two nonrandomized studies (NRSs)^25^^,^^26^; five cRCTs provided data on both *P. falciparum* and *P. vivax*,^27^^,^^28^ and an additional seven studies provided data on *P. vivax* only (all NRSs, before–after studies; although one study in Kenya looked at both *P. falciparum* and *P. vivax*, only data on *P. vivax* was included because the study did not meet inclusion criteria for *P. falciparum*).^22^^,^^29^^–^^34^ After identifying a high degree of heterogeneity (*I*^2^ = 90%) in parasitemia prevalence at 1 to 3 months, studies reporting data on *P. falciparum* were stratified into areas of moderate to high (≥10%) versus very low to low (<10%), which reduced the heterogeneity of pooled effects to an acceptable level (*I*^2 ^<60%). Because clusters in the Zambia trial were randomized a priori within low and high malaria transmission strata by design, we considered this trial as two studies.^21^ One study included multiple countries: Viet Nam, Cambodia, Laos, and Myanmar.^28^ Because there were differences in the timing, bias, and available outcomes, each site was analyzed as a separate study. Additionally, an article from the Solomon Islands reported two unique studies.^33^ Descriptive characteristics of each study reporting empirical data are summarized in Table 1 and Supplemental Table 3.

**Table 1:** Summary characteristics of included studies

Study (location, years of study)	Study Design	MDA		Outcomes Reported (months of follow-up post-MDA)
		Rounds, Interval, Duration	Population targeted (coverage)	
Cluster randomized trials				
von Seidlein 2003^35^ (Gambia, 1999)	cRCT	All persons ≥6 months old; pregnant women excluded; one round SP+AS in June 1999 Transmission season: June–December	3,655 (89%)	• *Pf *parasitemia prevalence (high transmission): 4–12 months
Shekalaghe 2011^23^ (Zanzibar, 2008)	cRCT	Ages >1 year, SP+AS with single dose PQ (0.75 mg/kg); one round; 16 days during January 2008–July 2008 Transmission season: March–May, October–November	1,110 (94.6%)	• *Pf* parasitemia prevalence (low transmission): 0–1, 4–12 months
Morris 2018^22^ (Zanzibar, 2016-2017)	cRCT	All ages >6 months; DP plus single low-dose PQ; two rounds every 4 weeks, April 2016–June 2016 Transmission season: April–August	10,944 (91%–80%)	• *Pf* parasitemia prevalence (low transmission): 1–3 months • *Pf* clinical malaria incidence (low transmission): 4–12, 12–24 months
von Seidlein 2019^28^ (Viet Nam, 2013–2014)	cRCT	All ages ≥6 months; 3 monthly rounds of DP + single low-dose PQ (0.25 mg/kg); November 2013, January–February 2014 Transmission season: May–November	1,439 (83% R1; 98% R2; 99% R3)	• Parasitemia prevalence (*Pv *and *Pf *low transmission): 1–3, 4–12 months • Confirmed malaria illness incidence (*Pv* and *Pf* low transmission): 4–12 months
Myanmar (2013–2014)		All ages ≥6 months; 3 monthly rounds of DP + single low-dose PQ (0.25 mg/kg); May–July 2013 or June–August 2013 Transmission season: June–October	1,434 (66% R1; 56% R2; 65% R3)	
Laos (2016–2017)		All ages ≥6 months; 3 monthly rounds of DP + single low-dose PQ (0.25 mg/kg); April, June, and July 2016 Transmission season: May–October	1,006 (81% R1; 80% R2; 82% R3)	
Cambodia (2014–2016)		All ages ≥6 months; 3 monthly rounds of DP; July–September 2015 Transmission season: May–October	858 (74% R1; 60% R2; 71% R3)	
Eisele 2020A^21^ (Zambia, 2014–2017) (Low transmission)	cRCT	MDA to all nonpregnant persons aged > 3 months with DP; four rounds over 15 months from December 2014–March 2016; low transmission area Transmission season: April–May	37,694 (79% R1, 63% R2, 76% R3, 66% R4)	• *Pf* parasitemia prevalence: 1–3 months • *Pf *parasitemia incidence: 1–3 months • *Pf *clinical malaria incidence: 1–3 months
Eisele 2020B^21^ (Zambia, 2014–2017) (High transmission)	cRCT	MDA to all nonpregnant persons aged > 3 months; DP; four rounds over 15 months from January 2013 to May 2015; high transmission area Transmission season: April–May	45,442 (79% in R1, 63% in R2, 76% in R3, and 66% in R4)	• *Pf *parasitemia prevalence: 1–3 months • *Pf *parasitemia incidence: 1–3 months • *Pf *clinical malaria incidence: 1–3 months
McLean 2021^27^ (Myanmar, 2014–2017)	cRCT	DP plus single low-dose PQ (0.25 mg/kg); three rounds between March–May 2015 (during dry season) Transmission season: June–August	8,721 (90%–86%)	• Parasitemia prevalence—*Pv *and *Pf* low transmission: 0–1, 1–3, 4–12, 12–24 months
Nonrandomized studies				
Simeons 1938^34^ (India, 1935)	Uncontrolled before-and-after study	MDA administered to all persons; one round atebrin IM 300 mg daily for 2 days and plasmochin simplex 60 mg daily for 3 days; May–June 1935 Transmission season: March–August	5,650 (100%)	• *Pv* clinical malaria incidence 0–1, 1–3, 4–12 months
Jones 1958^32^ (Kenya, 1952–1953)	Uncontrolled before-and-after study	MDA administered to all persons with pyrimethamine 100 mg once; three rounds in September 1952, March 1953, and September 1953 Transmission season: January–March, May–August	3,721 (Coverage not specified)	• *Pv *parasitemia prevalence: 1–3 months (Note: did not meet inclusion criteria for *Pf*, thus only *Pv *data are included)
Gabaldon 1959^31^ (Venezuela, 1956–1957)	Uncontrolled before-and-after study	MDA to all persons aged > 1 month with pyrimethamine 50 mg per week for 24 weeks from July 1957 to December 1957 Transmission season: May–November	111,995 (Coverage not specified)	• *Pv* parasitemia prevalence: 4–12 months • *Pv* parasitemia incidence 0–1, 1–3, 4–12 months
Schneider 1961^26^ (Burkina Faso, 1960–1961)	Non-RCT	Intervention group 1: MDA administered to all persons with a combination of 600- mg base CQ or AQ and 15-mg base PQ every 14 days in June–December 1960 for 15 rounds Intervention group 2: MDA administered to all persons with 600 mg base AQ and 15 mg base PQ every 14 days in June to December 1960 for eight rounds Comparison group 1: control zone free of any intervention (house spraying or treatment) Transmission season: August–September	6,035 (90% in intervention 1, 2,500 people; coverage not specified in intervention arm 2, 3,535 people)	• *Pf* parasitemia prevalence (high transmission): 1–3 months
Comer 1971^30^ (Panama, 1965–1968)	Uncontrolled before-and-after study	MDA to all persons aged >6 months. pyrimethamine 50 mg (cycles 1–25)/ 75 mg (cycles 26–49) and PQ 40 mg given every 2 weeks for 2 years from August 1966 to April 1968	1,548–1,908 (61–87%)	• Adverse outcomes
Paik 1974A^33^ (Solomon Islands, 1972)	Uncontrolled before-and-after study	MDA administered to all persons with CQ 600 mg + pyrimethamine 50 mg monthly for 4 months from July to October 1972 Transmission season: rainy season, December–April	Not specified (90%)	• *Pv* parasitemia prevalence: 0–1, 1–3, 4–12 months
Paik 1974B^33^ (Solomon Islands, 1972–1973)	Uncontrolled before-and-after study	MDA administered to all persons; 3 rounds CQ 1500 mg and PQ 75 mg every three months from October 1972 to March 1973 Transmission season: rainy season, December–April	1,200 (90%)	• *Pv* parasitemia incidence 0–1, 1–3 months
Molineaux 1980^25^ (Nigeria, 1970–1976)	Controlled before-and-after study	MDA to all persons: *low frequency—*SP as a single dose; nine rounds, every 10 weeks, 18 months April 1972–October 1973; *high frequency—*SP as a single dose; 23 rounds, every 2 weeks May–October 1972 and 1973 and every 10 weeks December 1972, March 1973, October–November 1973 Transmission season: April–October	14.129 (73–92%)	• *Pf* parasitemia prevalence- high transmission • 4–12, 12–24 months
Cáceres Garcia 2008^29^ (Venezuela, 2002–2007)	Uncontrolled before-and-after study	MDA to all nonpregnant persons aged > 6 months with CQ 25 mg/kg administered over 3 days and PQ 3.5 mg/kg in November 2002 Transmission season: November	25,722 (77% (of census)/86% (of included)	• *Pv* clinical malaria incidence 0–1, 1–3 months

AQ = amodiaquine; AS = artesunate; CQ = chloroquine; cRCT = cluster-randomized trial; DP = dihydroartemisinin piperaquine; IM = intramuscular; MDA = mass drug administration; *Pf *= *Plasmodium falciparum*; PQ = primaquine; *Pv* = *Plasmodium vivax*; R1–R4 = rounds 1–4; SP = sulfadoxine-pyrimethamine.

Ten cRCTs assessed the effects of MDA on *P. falciparum*. Two used sulfadoxine-pyrimethamine + artesunate (SP+AS), either alone^24^ or in combination with single low dose of primaquine (PQ; 0.75 mg/kg).^23^ Four used dihydroartemisinin piperaquine (DP) alone.^21^^,^^28^ Four used DP plus a single low dose of PQ (0.25 mg/kg)^27^^,^^28^ and provided data on both *P. falciparum* and *P. vivax*.

The certainty of evidence for the majority of outcomes was low or very low (Table 2; Supplemental Table 4). There was moderate certainty for the effect of MDA on the incidence of *P. falciparum* parasitemia at 1 to 3 months (both strata: low–very low and moderate–high transmission) and prevalence of *P. falciparum* parasitemia at 1 to 3 months in low–very low transmission settings.

**Table 2:** GRADE summary of findings tables for malaria outcomes

Outcomes	Studies and Participants	Rate Ratio or Relative Risk (95% CI)	Anticipated Absolute Effects (95% CI)		Certainity
			Risk with No MDA	Risk with MDA	
*Plasmodium falciparum *moderate/high transmission					
Incidence of parasitemia, 1–3 months	1 RCT 820.25 person-years	0.61 (0.40–0.92)	57 per 1,000 person-years	35 per 1,000 (23–52)	Moderate^‡^
Incidence of parasitemia, 4–12 months	1 RCT 517.75 person-years	0.91 (0.55–1.50)	108 per 1,000 person-years	98 per 1,000 (59–162)	Very low*^†^
Prevalence, 1–3 months	1 RCT 786 participants	1.76 (0.58–5.36)	50 per 1,000 person-years	88 per 1,000 (29–269)	Low*
Prevalence, 4–12 months	1 RCT 1,497 participants	1.18 (0.89–1.56)	483 per 1,000 person-years	570 per 1,000 (430–754)	Low*
Incidence of clinical malaria, 1–3 months	1 RCT 144,422 participants	0.41 (0.04–4.42)	2 per 1,000 person-years	1 per 1,000 (0–10)	Low*
*Plasmodium falciparum* low/very low transmission					
Incidence of parasitemia, 1–3 months	1 RCT 811.55 person-years	0.37 (0.21–0.66)	12 per 1,000 person-years	5 per 1,000 (3–8)	Moderate*
Prevalence, 0–1 months	2 RCTs 718 participants	0.12 (0.03–0.52)	35 per 1,000 person-years	4 per 1,000 (1–18)	Low*
Prevalence, 1–3 months	8 RCTs 6,511 participants	0.25 (0.15–0.41)	24 per 1,000 person-years	6 per 1,000 (4–10)	Moderate*
Prevalence, 4–12 months	6 RCTs 5,102 participants	0.82 (0.56–1.22)	19 per 1,000 person-years	16 per 1,000 (11–23)	Low*
Prevalence, 12–24 months	1 RCT 1,390 participants	0.34 (0.06–1.97)	32 per 1,000 person-years	11 per 1,000 (2–63)	Very low^§^^ǁ^^¶^
Incidence of clinical malaria, 1–3 months	2 RCTs 130,651 person-years	0.58 (0.12–2.73)	6 per 1,000 person-years	4 per 1,000 (1–17)	Low^†^
Incidence of clinical malaria, 4–12 months	4 RCTs 26,576 person-years	0.47 (0.21–1.03)	11 per 1,000 person-years	5 per 1,000 (2–12)	Very low*^‡^^§^
Incidence of clinical malaria, 12–24 months	1 RCT 23,251 person-years	0.77 (0.20–3.03)	17 per 1,000 person-years	13 per 1,000 (3–51)	Low^§^
*Plasmodium vivax*					
Prevalence, 0–1 months	1 RCT 243 participants	0.18 (0.08–0.40)	272 per 1,000 person-years	49 per 1,000 (22–109)	Moderate*
Prevalence, 1–3 months	5 RCTs 2,672 participants	0.15 (0.10–0.24)	133 per 1,000 person-years	20 per 1,000 (13–32)	Very low*^†^^‡^
Prevalence, 4–12 months	5 RCTs 6,255 participants	1.01 (0.87–1.18)	96 per 1,000 person-years	97 per 1,000 (84–113)	Low*^ǁ^
Prevalence, 12–24 months	1 RCT 243 participants	0.81 (0.44–1.48)	175 per 1,000 person-years	142 per 1,000 (77–259)	Low^§^
Incidence of clinical malaria, 4–12 months	1 RCT 3,325 person-years	1.38 (0.97–1.95)	41 per 1,000 person-years	57 per 1,000 (40–80)	Very low*^#^

GRADE = Grading of Recommendations, Assessment, Development and Evaluations; MDA = mass drug administration; RCT = randomized controlled trials.

* Downgraded by 2 due to risk of bias. Many risk of bias domains judged as high risk or not enough information to determine. High risk of bias due to confounding in both studies included for this outcome.

^†^ Not downgraded for inconsistency due to single-study result.

^‡^ Not downgraded for indirectness because evidence was judged to be sufficiently direct for the domains of population, intervention, comparison, direct comparison, and outcome.

^§^ Not downgraded for imprecision because lower and upper confidence limits indicate the same direction of effect.

^ǁ^ Not downgraded for inconsistency. Both studies provided the same direction and a similar magnitude (qualitatively) of effect.

^¶^ Downgraded by 2 due to indirectness. Side effects were not measured or reported in the control group, so evidence is only provided in the intervention population.

^#^ Not downgraded for imprecision because this criterion is not applicable for this outcome (no effect measure presented).

### *Plasmodium falciparum*: moderate to high transmission settings.

#### Incidence of parasitemia.

**Figure 3.**
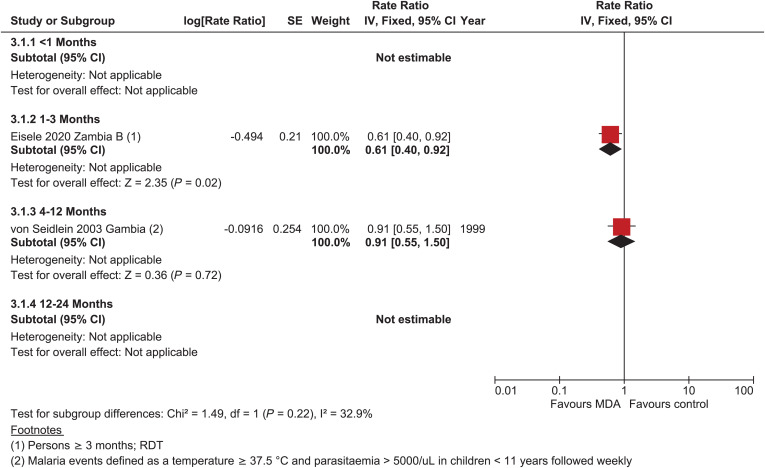
*Plasmodium falciparum* parasitemia incidence, cluster-randomized controlled studies, moderate–high transmission.

There were no data on the incidence of parasitemia within 1-month post-MDA in areas of moderate to high transmission. At 1 to 3 months post-MDA, there was a reduction in the incidence of parasitemia (rate ratio = 0.61, 95% CI: 0.40–0.92) of moderate certainty^21^; there was no difference in parasitemia incidence 4 to 12 months post-MDA (rate ratio = 0.91, 95% CI: 0.55–1.50), but the evidence was very uncertain (Figure 3).^35^ No study assessed the impact of MDA on the incidence of *P. falciparum* beyond 12 months post-MDA.

#### Prevalence of parasitemia.

**Figure 4.**
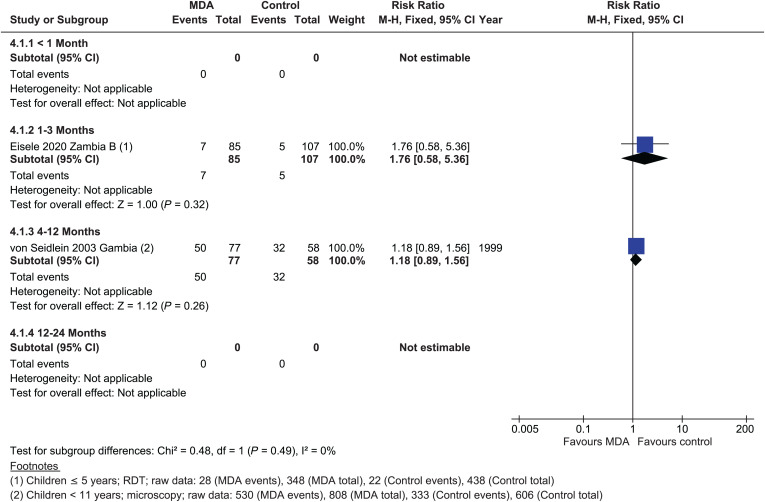
*Plasmodium falciparum*: parasitemia prevalence, cluster-randomized controlled studies, moderate–high transmission.

In the moderate to high transmission stratum, there were no studies within 1 month post-MDA measuring the impact on *P. falciparum* prevalence. No significant effect of MDA on the prevalence of *P. falciparum* parasitemia was detected 1 to 3 months post-MDA, with a single cRCT (risk ratio [RR] = 1.76, 95% CI: 0.58–5.36) (low certainty evidence),^21^ although a small effect was seen in a single NRS in an area of moderate to high transmission (RR = 0.85, 95% CI: 0.78–0.93)^26^ (Figure 4). Between 4 and 12 months post-MDA, the evidence suggests that MDA results in little to no difference in *P. falciparum* prevalence (RR = 1.18, 95% CI: 0.89–1.56).^35^ A single NRS suggested that MDA may slightly reduce *P. falciparum* parasitemia prevalence 4 to 12 months post-MDA (RR = 0.60, 95% CI: 0.55–0.67); this same study continued to show a very small benefit from 12 to 24 months (RR = 0.77, 95% CI: 0.70–0.84; Supplemental Figure 2).^25^

#### *Incidence of clinical *P. falciparum* malaria.*

There were no studies that assessed the incidence of *P. falciparum* clinical malaria within 1 month post-MDA. One cRCT provided data on the incidence of clinical malaria 1 to 3 months post-MDA in moderate–high settings^21^; there was a nonsignificant decrease in *P. falciparum* clinical malaria (rate ratio = 0.52, 95% CI: 0.04–1.91; Supplemental Figure 3). No studies assessed the incidence of clinical malaria at any of the subsequent time points.

### *Plasmodium falciparum*: Very low to low transmission settings.

#### Incidence of parasitemia.

**Figure 5.**
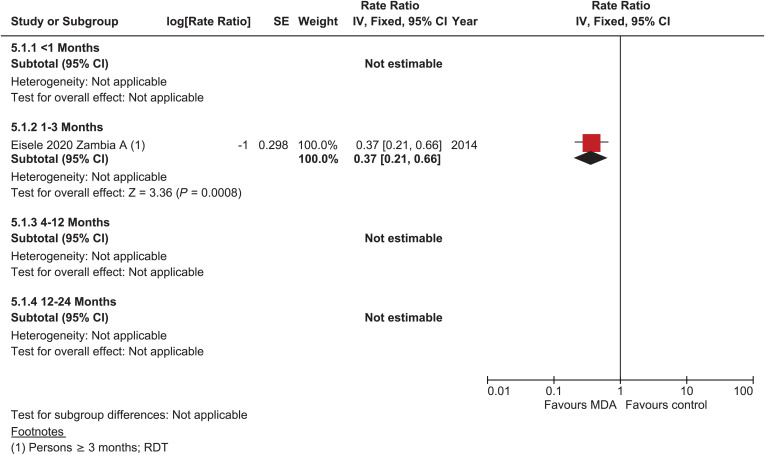
*Plasmodium falciparum*: parasitemia Incidence, cluster-randomized controlled studies, low–very low transmission.

One cRCT provided data on parasitemia incidence 1 to 3 months post-MDA in very low to low transmission settings (rate ratio = 0.37, 95% CI: 0.21–0.66; Figure 5).^21^ There were no data on incidence outcomes for any other time points.

#### Parasite prevalence.

**Figure 6.**
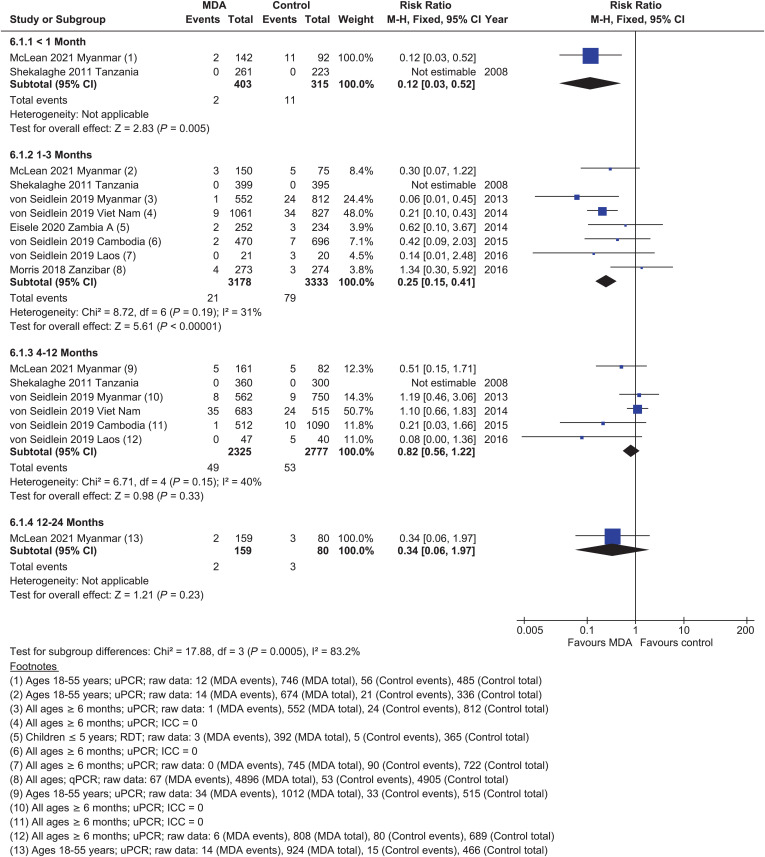
*Plasmodium falciparum*: parasitemia prevalence, randomized controlled trials, low–very low transmission.

Within 1 month post-MDA, there was a reduction in *P. falciparum* prevalence (RR = 0.12, 95% CI: 0.03–0.52).^27^ There was a large effect of MDA 1 to 3 months post-MDA on *P. falciparum* prevalence in very low to low transmission settings (RR = 0.25, 95% CI: 0.15–0.41, seven cRCTs; Figure 6).^21^^,^^22^^,^^27^^,^^28^ By 4 to 12 months post-MDA, the evidence from five cRCTs suggests that MDA results in little to no difference in *P. falciparum* prevalence (RR = 0.82, 95% CI: 0.56–1.22).^27^^,^^28^ Only one cRCT followed patients beyond 12 months, suggesting no significant benefit (RR = 0.34, 95% CI: 0.06–1.97).^27^

#### Incidence of clinical malaria.

There were no studies that assessed the incidence of *P. falciparum* clinical malaria within 1 month post-MDA. Two cRCTs^21^^,^^22^ provided data on the incidence of clinical malaria 1 to 3 months post-MDA in low–very low transmission settings. Mass drug administration resulted in a nonsignificant decrease in the incidence of *P. falciparum* clinical malaria (rate ratio = 0.58, 95% CI: 0.12–2.73, Supplemental Figure 4). A nonsignificant reduction in the incidence of clinical malaria was seen in a single study at 4 to 12 months (rate ratio = 0.47, 95% CI: 0.21–1.03) as well as at 12 to 24 months post-MDA (rate ratio = 0.77, 95% CI: 0.20–3.03).^22^

### Plasmodium vivax.

#### Incidence of parasitemia.

There were no cRCTs providing data on the impact of MDA on the incidence of *P. vivax* parasitemia. Two NRSs demonstrated an 85% reduction in the incidence of *P. vivax* within 1 month of MDA (rate ratio = 0.15, 95% CI: 0.12–0.19); the effect remained at 1 to 3 months (rate ratio = 0.37, 95% CI: 0.32–0.43) and 4 to 12 months (rate ratio = 0.15, 95% CI: 0.07–0.34), although the certainty of evidence was very low (Supplemental Figure 5).^31^^,^^33^

#### Parasite prevalence.

**Figure 7.**
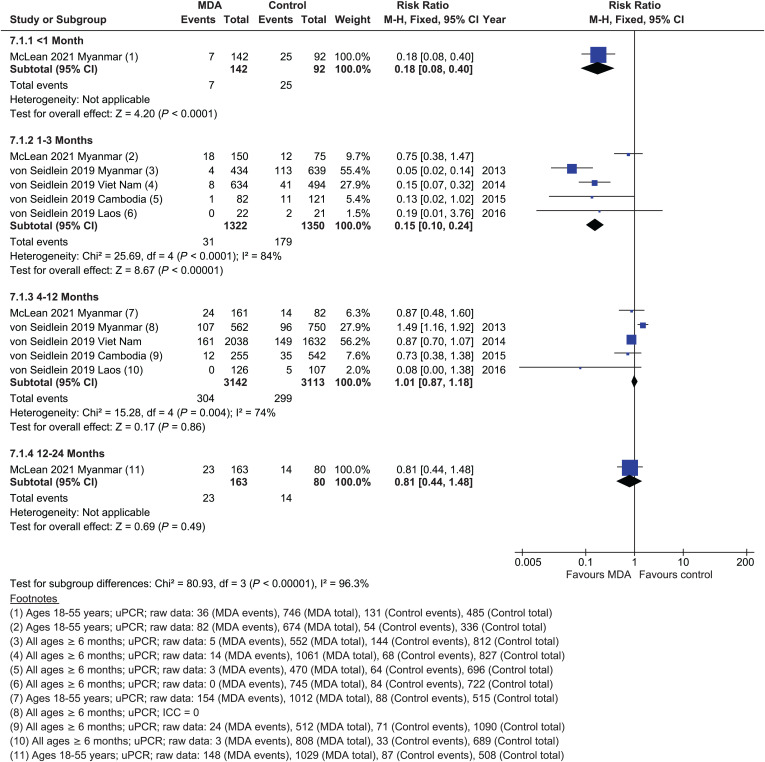
*Plasmodium vivax*: parasitemia prevalence, randomized controlled trials.

The prevalence of *P. vivax* was reduced within 1 month post-MDA (RR = 0.18, 95% CI: 0.08–0.40) (Figure 7)^27^; a reduction was also found at 1 to 3 months post-MDA (RR = 0.15, 95% CI: 0.10-0.24, five studies),^27^^,^^28^ but there was significant heterogeneity between studies (*I*^2^ = 84%) that could not be explained. There was no effect of MDA on *P. vivax* prevalence between 4 and 12 months (RR = 1.01, 95% CI: 0.87–1.18, five studies)^27^^,^^28^ or between 12 and 24 months (RR = 0.81, 95% CI: 0.44–1.48, single study).^27^ Reductions in parasite prevalence were identified in NRSs within 1 month post-MDA (RR = 0.32, 95% CI: 0.12–0.87),^33^ at 1 to 3 months (RR = 0.18, 95% CI: 0.10–0.33),^32^^,^^33^ and at 4 to 12 months (RR = 0.34, 95% CI: 0.15-0.78; Supplemental Figure 6).^33^

#### Incidence of clinical malaria.

Data on confirmed clinical malaria incidence with *P. vivax* derived from two cRCTs (Cambodia and Myanmar)^28^ that collected data between 4 and 12 months post-MDA demonstrated a nonsignificant increased incidence (rate ratio = 1.38, 95% CI: 0.97–1.95; Supplemental Figure 7).

Two NRSs demonstrated a reduced incidence of clinical malaria within 1 month post-MDA (rate ratio = 0.23, 95% CI: 0.21–0.31; Supplemental Figure 8) and 1 to 3 months post-MDA (rate ratio = 0.29, 95% CI: 0.26–0.31; Supplemental Figure 8).^29^^,^^36^ There was a reduction in clinical malaria incidence at 4 to 12 months (rate ratio = 0.72, 95% CI: 0.68–0.76) and 12–24 months post-MDA (rate ratio = 0.04, 95% CI: 0.02–0.07), although the certainty of evidence was low.^36^

### Adverse events and serious adverse events.

#### Plasmodium falciparum.

Only the 2019 studies in Myanmar and Vietnam measured serious adverse events (SAEs) in both the MDA and control arms.^28^ There was a nonsignificant increase in the odds of an SAE in the MDA arm (odds ratio [OR] = 3.61, 95% CI: 0.43–30.03 for 0–3 months post-MDA and OR 1.47, 95% CI: 0.68–3.20 for 4–12 months post-MDA; Supplemental Figure 9). None of the SAEs were determined to have been related to MDA. Another four studies reported on SAEs in the MDA arm only^21^^–^^23^^,^^27^; there were a total of 12 reported SAEs out of a total of 353,143 doses of MDA, or a rate of SAEs of three per 100,000 doses. Only two of these SAEs were considered potentially related to MDA: one serious skin reaction^23^ and one SAE that was not well described.^21^

Only one study reported numbers of AEs in both MDA and placebo arms, although this was limited to a proportion of the study population.^24^ One-third (25) of 75 individuals remembered at least one complaint within 2 days of MDA, compared with two of 15 individuals (13%) who had received placebo (OR = 3.25, 95% CI: 0.68–15.53). Among those who received MDA, complaints included dizziness (17%), fever (8%), diarrhea (7%), vomiting (7%), and itching (5%). Among the other four studies that reported AEs in the MDA arm only,^21^^,^^22^^,^^27^^,^^37^ AEs occurred at a rate of 4.6 per 1,000 doses (1734 AEs out of 376,807 doses).

Among one cRCT, vomiting following MDA with SP+AS with or without PQ was not significantly different in the MDA versus placebo arm (OR = 0.54, 95% CI: 0.19–1.54).^23^^,^^38^

#### Plasmodium vivax.

Two MDA studies reported AEs from areas with *P. vivax* transmission; there were 749 AEs and 12 SAEs among 37,575 courses of MDA, a rate of 19.9 AEs per 1,000 courses and 0.32 SAEs per 1,000 courses.^27^^,^^28^ One study noted “individual complaints of nausea and headache without observing any severe secondary effects,” but no exact number was provided; none of those who complained about an AE refused subsequent rounds.^30^

### Antimalarial resistance.

A study in Burma (Myanmar) identified *PfKelch13* mutations in 28 (54%) of 52 and 27 (64%) of 42* P. falciparum* positive samples at baseline in the MDA arm and control arm, respectively, from among 621 individuals sampled in the MDA arm and 412 in the control arm (Supplemental Figure 10).^27^ At month 3 after MDA, six of 12 (50%) of 747 individuals sampled in the MDA arm, and 31 of 51 (61%) of 485 sampled individuals in the control arm harbored parasites with the *PfKelch13* mutation (Supplemental Figure 11); 57% of people in the MDA arm with symptomatic malaria had not received MDA. At month 21, seven (64%) and 10 (71%) of samples in the MDA and control arms, respectively, had the *PfKelch13* mutation, and by month 27, this was 14 (70%) and 11 (85%). All individuals with asymptomatic *P. falciparum* (*N* = 57) or *P. vivax* (*N* = 93) infections at baseline who received a third round of MDA were negative on retesting. No samples with multi-copy *pfplasmepsin2–3* were found.

### Potential effect modifiers.

The number of studies eligible for inclusion in this review was too few to permit stratification of the metanalysis by variables other than transmission level. The cRCTs assessed between one and four rounds of drug, whereas the NRSs included one to 52 rounds (every 2 weeks for 2 years), most of which were distributed either immediately before or during the transmission season. One cRCT (the Viet Nam site of von Seidlein et al.) and two NRSs (an uncontrolled before-and-after study in Kenya) administered drug starting at the end of the transmission season or in dry season.^28^^,^^32^^,^^33^ Coverage of MDA among the cRCTs varied from 56% in Myanmar to 99% in Viet Nam^28^ and from 61% to 100% where specified in the NRS.^30^^,^^36^ All but one of the eligible cRCTs included in the review were conducted in areas where insecticide-treated nets (ITNs) had been distributed; reported ITN use ranged from 25.1%^23^ to 84.4%^28^; IRS was also deployed for vector control in three of the cRCTs (Morris, Eisele A and Eisele B) with household coverage ranging from 6.9% to 85% (Supplemental Table 3).^21^^,^^22^ Among the eight NRSs, four reported IRS in the areas where the studies took place,^25^^,^^26^^,^^31^^,^^33^ but coverage was measured and reported in only one.^25^ Among the NRS, many of which were conducted before 2000, no ACTs were used.^25^^,^^26^^,^^29^^–^^33^^,^^36^ Single low-dose primaquine (0.25 mg/kg), which is recommended to kill gametocytes of *P. falciparum*, was included in five of the 10 cRCTs, including the Viet Nam, Myanmar, and Laos sites of von Seidlein 2019,^22^^,^^23^^,^^27^^,^^28^ and a sixth cRCT included a single 0.75 mg/kg primaquine gametocytocidal dose. Among the three NRSs that included primaquine, two assessed the incidence or prevalence of *P. vivax* as an outcome, and only one used a higher dosage of primaquine that would have also killed hypnozoites.^26^ Only two of the cRCTs and two of the NRSs were conducted on islands (Zanzibar and the Solomon Islands) but even in these studies, communities receiving MDA were not isolated from untreated communities.^22^^,^^23^ The degree of geographic isolation of communities participating in MDA in the majority of studies could not be determined.

## DISCUSSION

There is renewed interest in MDA as a potential intervention to accelerate elimination of malaria; this review assessed the effects of MDA on transmission of both *P. falciparum* and *P. vivax* to provide the WHO with the information needed to develop species-specific recommendations on MDA. From a theoretical standpoint, administering antimalarials to an entire population should have a substantial impact on the prevalence and incidence of malaria by clearing the human infectious reservoir and preventing new infections for a period of time. However, the evidence from multiple studies demonstrates that the impact of MDA is short-lived. Overall, MDA had a significant effect on *P. falciparum* incidence within 1 to 3 months post-MDA in both very low–low and moderate–high transmission settings, although the effect was greater in very low–low transmission settings. Because of significant heterogeneity in this outcome between studies in very low–low and moderate–high transmission settings, the impact of MDA on *P. falciparum* was meta-analyzed separately in these two strata. In very low–low, but not moderate–high, transmission settings, there was also a significant effect on the prevalence of *P. falciparum* up to 3 months post-MDA, but MDA appears to have little to no effect on the incidence of clinical malaria due to *P. falciparum* (low certainty evidence). By 4 to 12 months post-MDA and beyond, MDA likely results in little to no difference in the incidence or prevalence of *P. falciparum* in either transmission strata.

For *P. vivax,* the evidence suggests that MDA may reduce prevalence at 1 to 3 months post-MDA, although the certainty of the evidence is low with a very high degree of heterogeneity (*I*^2^ = 84%). For subsequent time points, it is unclear whether MDA provides any benefit because there were few cases of malaria, and the evidence was of low or very low certainty. There was no impact of MDA on the prevalence of *P. vivax* at 4 to 12 months among the cRCTs, although a single NRS demonstrated a significant impact on *P. vivax* prevalence at 4 to 12 months post-MDA. Although evidence from cRCTs did not demonstrate a significant impact of MDA on the incidence of *P. vivax* parasitemia or clinical malaria, data from NRSs showed statistically significant reductions at several time points; however, the certainty of this evidence was very low. Among all the studies, only one NRS, that of Cáceres Garcia in 2008,^29^ provided a sufficient dose of primaquine for radical cure, limiting the ability to determine whether the administration of radical cure affected longer term outcomes.

Overall, the available data suggest that providing a limited number of MDA rounds is likely to have only a short-term impact on malaria parasitemia prevalence. However, given the limited number of studies that met the review inclusion criteria and the differences in study design (differing time points, outcomes, and drugs), there were few studies available to identify factors that may modify the impact of MDA (e.g., coverage levels, number of rounds, or type of drug). Additionally, although several of the nonrandomized trials assessed more rounds, none of the RCTs examined more than four rounds of MDA spanning a period of more than 15 months, and even among the nonrandomized trials, the longest intervention period was 2 years, limiting our ability to evaluate the impact of a longer duration of intervention. Future studies to evaluate the effect of MDA on malaria should attempt to use more rigorous study designs (e.g., quasi-experimental cRCTs with a minimum of two clusters per arm, controlled before-and-after studies with a contemporaneous control group and at least two sites per arm, or interrupted time series with at least three data points before and after the intervention) and standardized outcome measurements.

## CONCLUSION

Mass drug administration may have short-term benefits for incidence and prevalence of malaria that make it useful in some settings or contexts, particularly in lower transmission settings; however, we found no evidence for longer term impact, although none of the trials provided data on prolonged interventions. The context and goals of MDA should be carefully considered before deciding to implement to assess the appropriateness of the intervention in the specific context.

## Supplemental Materials

10.4269/ajtmh.22-0766 Supplemental Materials
