# Supplementary material for: Mass Drug Administration to Reduce Malaria Transmission: A Systematic Review and Meta-Analysis
Source: Am J Trop Med Hyg. 2023 Dec 20;110(4 Suppl):17–29. doi: 10.4269/ajtmh.22-0766 (PMC10993786; doi:10.4269/ajtmh.22-0766)

Supplementary Data

Mass drug administration for malaria transmission reduction: A systematic review and meta-analysis

Zachary D. Schneider, Monica P. Shah, Marisa C. Boily, Alexandra L. Busbee, Jimee Hwang, Kim A. Lindblade, Julie R. Gutman

Supplementary Table 1. Search strategy

Search Name: Cochrane Central Register of Controlled Trials

ID	Search
#2	"antimalarial":ti,ab,kw (Word variations have been searched)
#3	malaria:ti,ab,kw (Word variations have been searched)
#4	MeSH descriptor: [Malaria] explode all trees
#5	MeSH descriptor: [Antimalarials] explode all trees
#6	#2 or #3 or #4 or #5
#7	"mass chemoprophylaxis" or "mass drug administration" or "mass administration"
#8	"mass screening and treatment"
#9	"mass treatment"

Database: Embase 1947-Present, updated daily

1	malaria/ or malaria control/
2	antimalarial agent/ or antimalarial*.mp.
3	(malaria or antimalarial*).ab. or (malaria or antimalarial*).ti.
4	1 or 2 or 3
5	("mass chemoprophylaxis" or "mass drug administration" or "mass administration").mp.
6	mass drug administration.mp.
7	mass treatment.mp.
8	"mass screening and treatment".mp.
9	5 or 6 or 7 or 8
10	4 and 9

Pubmed Search history

Search	Query
#1	malaria Field: Title/Abstract
#2	antimalarial*or anti-malarial* Field:Title/Abstract
#3	(malaria[MeSH Terms]) OR antimalarials[MeSH Terms]
#4	#1 or #2 or #3
#5	((mass chemoprophylaxis) OR mass drug administration) OR mass administration

	Field:Title/Abstract
#6	"mass screening and treatment" Field: Title/Abstract
#7	(MDA[Title/Abstract] OR MSAT[Title/Abstract] OR iMSaT[Title/Abstract])
#8	mass drug administration[MeSH Terms]
#9	#5 or #6 or #7 or #8
#10	#4 and #9

Database : **LILACS**

Search on:	**malaria or antimalarial$ [Words] and mass administration [Words]**

Supplementary Table 2. List of studies excluded after full review and primary reasons for exclusion

Study	#	REASON FOR EXCLUSION	Title	Citation
Bennett 2020	722	Cross reference with outcomes; Linked to Eisele 2020 (#723)	A Longitudinal Cohort to Monitor Malaria Infection Incidence during Mass Drug Administration in Southern Province, Zambia	2020. AJTMH;103(2_Suppl):54-65
Bennett 2016	61	Duplicate; ASTMH abstract - duplicate of #722; Linked to Eisele 2020 (#723)	A longitudinal cohort to monitor malaria infection incidence in the context of a community randomized trial of mass drug administration in Southern Province, Zambia	2016. AJTMH;95 (5 Supplement 1)():489
Chishimba 2020	728	No outcomes reported; Linked to Eisele 2020 (#723)	Prevalence of Plasmodium falciparum and Non-falciparum Infections by Photo-Induced Electron Transfer-PCR in a Longitudinal Cohort of Individuals Enrolled in a Mass Drug Administration Trial in Southern Province, Zambia	2020. AJTMH;103(2_Suppl):82-89
Daniels 2020	729	No outcomes reported; Linked to Eisele 2020 (#723)	Evidence for Reduced Malaria Parasite Population after Application of Population-Level Antimalarial Drug Strategies in Southern Province, Zambia	2020. AJTMH;103(2_Suppl):66-73
Finn 2016	60	Duplicate; ASTMH abstract; Linked to Eisele 2020 (#723)	Treatment adherence to dihydroartemisinin-piperaquine during mass drug administration for malaria in Southern Province, Zambia	2016. AJTMH;95 (5 Supplement 1)():284
Finn 2020	727	Cross reference with additional details; Linked to Eisele 2020 (#723)	Treatment Coverage Estimation for Mass Drug Administration for Malaria with Dihydroartemisinin-Piperaquine in Southern Province, Zambia	2020. AJTMH;103(2_Suppl):19-27
Finn 2020	731	Cross reference with additional details; Linked to Eisele 2020 (#723)	Adherence to Mass Drug Administration with Dihydroartemisinin-Piperaquine and Plasmodium falciparum Clearance in Southern Province, Zambia	2020. AJTMH;103(2_Suppl):37-45
Galactionova 2020	713	Provides Contextual Factors; linked to Eisele 2020 (723)	Costing malaria interventions from pilots to elimination programmes	2020. Malaria journal;19(1):332
Miller 2020	730	No outcomes reported; linked to Eisele 2020 (723)	Moving from Malaria Burden Reduction toward Elimination: An Evaluation of Mass Drug Administration in Southern Province, Zambia	2020. AJTMH;103(2_Suppl):44261
Porter 2020	726	Contextual Factors; linked to Eisele 2020 (723)	Recent Travel History and Plasmodium falciparum Malaria Infection in a Region of Heterogenous Transmission in Southern Province, Zambia	2020. AJTMH;103(2_Suppl):74-81
Scott 2015	36	ASTMH abstract - duplicate of #725; Linked to Eisele 2020 (#723)	Evaluating the costs of implementing a package of interventions and surveillance systems to support malaria elimination in southern province, zambia: A micro-costing analysis	2015. AJTMH;93 (4 Supplement)():268
Silumbe 2020	720	Provides contextual Factors only; linked to Eisele 2020 (723)	Assessment of the Acceptability of Testing and Treatment during a Mass Drug Administration Trial for Malaria in Zambia Using Mixed Methods	2020. AJTMH;103(2_Suppl):28-36
Steketee 2020	724	Cross reference with outcomes; linked to Eisele 2020 (723)	Implications of the MDA Trial in Southern Province, Zambia, for Malaria Control and Elimination	2020. AJTMH;103(2_Suppl):98-101
Yukich	725	Contextual Factors; linked to Eisele 2020 (723)	Cost-Effectiveness of Focal Mass Drug Administration and Mass	2020.

2020			Drug Administration with Dihydroartemisinin-Piperaquine for Malaria Prevention in Southern Province, Zambia: Results of a Community-Randomized Controlled Trial	AJTMH;103(2_Suppl):46-53
Anonymous 2017	128	No outcomes reported; linked to Eisele 2020 (723)	Erratum: Short-term impact of mass drug administration with dihydroartemisinin plus piperaquine on malaria in southern province zambia: A cluster-randomized controlled trial	2017. J Inf Dis;216(8):1048
Bever 2016	67	Contextual Factors; ASTMH abstract; linked to Eisele 2020 (723)	Epidemiological and operational lessons learned from a malaria elimination campaign in zambia's lake kariba region	2016. AJTMH;95 (5 Supplement 1)():490
Chalwe 2016	57	Cross reference with outcomes; ASTMH abstract; linked to Eisele 2020 (723)	Adverse event reporting from malaria mass drug administration rounds conducted in southern zambia	2016. AJTMH;95 (5 Supplement 1)():487
Chiyende 2016	79	Cross reference with outcomes; ASTMH abstract; linked to Eisele 2020 (723)	Targeted community sensitization to reduce anticipated refusals in malaria mass drug administration trial: Lessons learned from Southern Zambia	2016. AJTMH;95 (5 Supplement 1)():485
Conner 2017	112	No outcomes reported; ASTMH abstract; linked to Eisele 2020 (723)	Programmatic mass drug administration in southern province, Zambia: An evaluation of impact and possible spill-over effects using dhis2 malaria case incidence data	2017. AJTMH;97 (5 Supplement 1)():501
Dieye 2017	111	Contextual Factors; linked to Eisele 2020 (723)	Malaria elimination: Engaging communities through nationwide campaigns	2017. AJTMH;97 (5 Supplement 1)():592
Eisele 2015	448	Cross reference with additional details; linked to Eisele 2020 (723)	Assessing the effectiveness of household-level focal mass drug administration and community-wide mass drug administration for reducing malaria parasite infection prevalence and incidence in Southern Province, Zambia: study protocol for a community randomi	2015. Trials;16():347
Eisele 2015	40	Duplicate; ASTMH abstract - duplicate of #488; linked to Eisele 2020 (723)	The impact of targeted mass drug administration using dihydroartemisinin-piperaquine in southern province Zambia: Initial findings	2015. AJTMH;93 (4 Supplement)():83
Eisele 2016	488	Cross reference with outcomes; linked to Eisele 2020 (723)	Short-term Impact of Mass Drug Administration With Dihydroartemisinin Plus Piperaquine on Malaria in Southern Province Zambia: A Cluster-Randomized Controlled Trial	2016. J Inf Dis;214(12):1831-1839
Eisele 2017	108	Duplicate; ASTMH abstract, linked to Eisele 2020 (723)	The long-term durability of mass drug administration using dihydroartemisinin-piperaquine as part of a comprehensive malaria elimination strategy in Southern province zambia	2017. AJTMH;97 (5 Supplement 1)():309
Galactionova 2019	179	Duplicate; linked to Eisele 2020 (723)	From pilots to an elimination program: How much do malaria interventions cost	2019. AJTMH;101 (5 Supplement)():512-513
Nct 2014	275	Cross reference with additional details; linked to Eisele 2020 (723)	Mass Drug Administration With Dihydroartemisinin + Piperaquine for Reducing Malaria in Southern Zambia	2014. https://clinicaltrials.gov/show/NCT02329301;():
Silumbe 2016	81	Contextual Factors; ASTMH abstract; linked to Eisele 2020 (723)	Transitioning an evidence-based malaria mass drug administration (MDA) research strategy to program/routine mode: Factors for consideration	2016. AJTMH;95 (5 Supplement 1)():487
Silumbe 2019	173	Duplicate; ASTMH abstract- data duplicated in #723; linked to Eisele 2020 (723)	Reductions in malaria burden through the use of a scalable intervention package (SIP) in accordance with the Zambia	2019. AJTMH;101 (5 Supplement)():524

			national malaria elimination strategic plan 2017-2021: The case of Mulobezi district in Western Province	
Stuckey 2016	467	Modelling data; linked to Eisele 2020 (723)	Operational strategies of anti-malarial drug campaigns for malaria elimination in Zambia's southern province: a simulation study	2016. Malaria journal;15():148
Suresh 2018	139	No outcomes reported; ASTMH abstract; linked to Eisele 2020 (723)	Stratification of malaria transmission dynamics and optimal intervention packages in Zambia and Mozambique	2018. AJTMH;99 (4 Supplement)():357-358
Suresh 2019	190	No outcomes reported; linked to Eisele 2020 (723)	Choosing the right tool for the job: Estimating effect sizes for multiple overlapping interventions in Southern Province, Zambia	2019. AJTMH;101 (5 Supplement)():416
Wenger 2013	384	No outcomes reported; ASTMH abstract ; linked to Eisele 2020 (723)	Modeling for malaria control and elimination scenario planning: Application of the epidemiological modeling (EMOD) malaria disease transmission kernel to community-based intervention delivery in Southern Zambia	2013. AJTMH;1)():396
Wenger 2014	401	No outcomes reported; linked to Eisele 2020 (723)	Spatial dynamics of malaria transmission in the EMOD model for campaigns targeting sustained regional elimination in Southern Zambia	2014. AJTMH;1)():14
Fraser 2020	739	Design: insufficient data points for ITS; ITS analysis of programmatic rounds of MDA. However, IRS partially implemented at the same time as MDA, so insufficient data points to evaluate MDA alone;	Evaluating the impact of programmatic mass drug administration for malaria in Zambia using routine incidence data	2020. J Inf Dis;():
Mancuso 2019	189	Cross reference with outcomes; linked to only #739 (Fraser 2020); ASTMH abstract	Ongoing assessment of plasmodium falciparum parasite prevalence in southern province Zambia: Results from a 2019 parasite survey three years after a mass drug administration trial	2019. AJTMH;101 (5 Supplement)():216
Adhikari 2017	526	Contextual Factors; linked to vonSeidlein 2019 (596)	Elements of effective community engagement: lessons from a targeted malaria elimination study in Lao PDR (Laos)	2017. Global health action;10(1):1366136
Adhikari 2017	530	Contextual Factors; linked to vonSeidlein 2019 (596)	Factors associated with population coverage of targeted malaria elimination (TME) in southern Savannakhet Province, Lao PDR	2017. Malaria journal;16(1):424
Adhikari 2018	538	Contextual Factors; linked to vonSeidlein 2019 (596)	Why do people participate in mass anti-malarial administration? Findings from a qualitative study in Nong District, Savannakhet Province, Lao PDR (Laos)	2018. Malaria journal;17(1):15
Adhikari 2018	593	Contextual Factors; linked to vonSeidlein 2019 (596)	Perceptions of asymptomatic malaria infection and their implications for malaria control and elimination in Laos	2018. PloS one;13(12):e0208912
Chaumeau 2019	616	Cross reference with outcomes; linked to vonSeidlein 2019 (596)	Contribution of Asymptomatic Plasmodium Infections to the Transmission of Malaria in Kayin State, Myanmar	2019. J Inf Dis;219(9):1499-1509
Imwong 2020	199	Cross reference with outcomes; linked to vonSeidlein 2019 (596)	Molecular epidemiology of resistance to antimalarial drugs in the Greater Mekong subregion: an observational study	2020. The Lancet Infectious Diseases.;():
Kajeechiwa 2016	482	Contextual Factors; linked to vonSeidlein 2019 (596)	The acceptability of mass administrations of anti-malarial drugs as part of targeted malaria elimination in villages along the Thai-Myanmar border	2016. Malaria journal;15(1):494
Kajeechiwa 2017	661	Contextual Factors; linked to vonSeidlein 2019 (596)	Community engagement for the rapid elimination of malaria: the case of Kayin State, Myanmar	2017. Wellcome open research;2():59
Landier 2016	72	Cross reference with outcomes; linked to vonSeidlein 2019 (596)	Relative contribution of generalized early diagnosis and treatment and of targeted mass treatment to elimination of	2016. AJTMH;95 (5 Supplement 1)():382

			plasmodium falciparum malaria in eastern myanmar	
Landier 2017	662	Cross reference with outcomes; linked to vonSeidlein 2019 (596)	Safety and effectiveness of mass drug administration to accelerate elimination of artemisinin-resistant falciparum malaria: A pilot trial in four villages of Eastern Myanmar	2017. Wellcome open research;2():81
Li 2019	186	No outcomes reported; linked to vonSeidlein 2019 (596)	Spatial analysis of parasite population genomics during malaria elimination efforts in Eastern Myanmar	2019. AJTMH;101 (5 Supplement)():597
Nct 2013	278	Cross reference with additional details; linked to vonSeidlein 2019 (596)	Targeted Chemo-elimination (TCE) of Malaria	2013. https://clinicaltrials.gov/show/NCT01872702;():
Nguyen 2017	497	Contextual Factors; linked to vonSeidlein 2019 (596)	Community perceptions of targeted anti-malarial mass drug administrations in two provinces in Vietnam: a quantitative survey	2017. Malaria journal;16(1):17
Parker 2019	598	Cross reference with outcomes; linked to vonSeidlein 2019 (596)	Potential herd protection against Plasmodium falciparum infections conferred by mass antimalarial drug administrations	2019. eLife;8():
Pell 2017	511	Contextual Factors; linked to vonSeidlein 2019 (596)	Mass anti-malarial administration in western Cambodia: a qualitative study of factors affecting coverage	2017. Malaria journal;16(1):206
Pell 2019	597	Contextual Factors; linked to vonSeidlein 2019 (596)	Community engagement, social context and coverage of mass anti-malarial administration: Comparative findings from multi-site research in the Greater Mekong sub-Region	2019. PloS one;14(3):e0214280
Peto 2018	537	Contextual Factors; linked to vonSeidlein 2019 (596)	Reflections on a Community Engagement Strategy for Mass Antimalarial Drug Administration in Cambodia	2018. AJTMH;98(1):100-104
Peto 2018	539	Contextual Factors; linked to vonSeidlein 2019 (596)	Community participation during two mass anti-malarial administrations in Cambodia: lessons from a joint workshop	2018. Malaria journal;17(1):53
Peto 2018	588	Contextual Factors; linked to vonSeidlein 2019 (596)	The feasibility and acceptability of mass drug administration for malaria in Cambodia: a mixed-methods study	2018. Trans Royal Soc Trop Med Hyg;112(6):264-271
Phommasone 2020	624	Cross reference with outcomes; linked to vonSeidlein 2019 (596)	Mass drug administrations with dihydroartemisinin-piperaquine and single low dose primaquine to eliminate Plasmodium falciparum have only a transient impact on Plasmodium vivax: Findings from randomised controlled trials	2020. PloS one;15(2):e0228190
Pongvongsa 2018	559	Cross reference with outcomes; linked to vonSeidlein 2019 (596)	The dynamic of asymptomatic Plasmodium falciparum infections following mass drug administrations with dihydroarteminisin-piperaquine plus a single low dose of primaquine in Savannakhet Province, Laos	2018. Malaria journal;17(1):405
Sahan 2017	500	Contextual Factors; linked to vonSeidlein 2019 (596)	Community engagement and the social context of targeted malaria treatment: a qualitative study in Kayin (Karen) State, Myanmar	2017. Malaria journal;16(1):75
Tangseefa 2018	705	Contextual Factors; linked to vonSeidlein 2019 (596)	"Nine Dimensions": A multidisciplinary approach for community engagement in a complex postwar border region as part of the targeted malaria elimination in Karen/Kayin State, Myanmar	2018. Wellcome open research;3():116
Tripura 2018	1	Cross reference with outcomes; linked to vonSeidlein 2019 (596)	A Controlled Trial of Mass Drug Administration to Interrupt Transmission of Multidrug-Resistant Falciparum Malaria in Cambodian Villages	2018. Clin Inf Dis;67(6):817-826

Tun 2017	533	No outcomes reported; linked to vonSeidlein 2019 (596)	Towards malaria elimination in Savannakhet, Lao PDR: mathematical modelling driven strategy design	2017. Malaria journal;16(1):483
vonSeidlein 2019	619	No outcomes reported; linked to vonSeidlein 2019 (596)	The probability of a sequential Plasmodium vivax infection following asymptomatic Plasmodium falciparum and P. vivax infections in Myanmar, Vietnam, Cambodia, and Laos	2019. Malaria journal;18(1):449
Mwesigwa 2019	631	Design: control group criteria not met; not designed as ITS; no control group and outcomes are pf	Mass Drug Administration With Dihydroartemisinin-piperaquine and Malaria Transmission Dynamics in The Gambia: A Prospective Cohort Study	2019. Clin Inf Dis;69(2):278-286
Mwesigwa 2016	74	Cross reference with outcomes; ASTMH abstract - full publication is #631	Impact of mass drug administration with dihydroartemisinin-piperaquine on malaria transmission in a highly seasonal transmission setting in the gambia	2016. AJTMH;95 (5 Supplement 1)():382
Mwesigwa 2017	103	Cross reference with outcomes; ASTMH abstract - full publication is Mwesigwa 2019 (#631)	Impact of two annual cycles of mass drug administration on temporal trends of clinical malaria	2017. AJTMH;97 (5 Supplement 1)():411
Mwesigwa 2018	152	Duplicate; ASTMH abstract - full publication is Mwesigwa 2019 (#631)	Impact of mass treatment with dihydroartemisinin piperaquine on malaria transmission dynamics in the Gambia: A prospective study	2018. AJTMH;99 (4 Supplement)():126
Mwesigwa 2019	609	No outcomes reported; full publication is Mwesigwa 2019 (#631)	Field performance of the malaria highly sensitive rapid diagnostic test in a setting of varying malaria transmission	2019. Malaria journal;18(1):288
Dierickx 2015	47	Duplicate; ASTMH abstract - full publication is #464; linked Mwesigwa 2019 (#631)	Community barriers for the implementation of a mass drugs administration for malaria in the Gambia	2015. AJTMH;93 (4 Supplement)():197
Dierickx 2016	464	Contextual Factors; linked to Mwesigwa 2019 (#631)	Factors Associated with Non-Participation and Non-Adherence in Directly Observed Mass Drug Administration for Malaria in The Gambia	2016. PloS one;11(2):e0148627
Dial 2014	671	Contextual Factors; linked to 631- Mwesigwa 2019 (631)	**A qualitative study to assess community barriers to malaria mass drug administration trials in The Gambia**	2014. Malaria journal;13():47
Siling 2016	73	Contextual Factors; ASTMH abstract; linked to Mwesigwa 2019 (631)	Ignoring people 'who are not there' may mitigate success of mass drug administration for malaria: Findings from a mixed-method study in the gambia	2016. AJTMH;95 (5 Supplement 1)():7
Wu 2018	136	No outcomes reported; ASTMH abstract	Validating novel serological markers of malaria exposure: Evaluating the effect of mass drug administration (MDA) and seasonal malaria chemoprevention (SMC) on transmission in rural Gambia based on population-level antibody responses	2018. AJTMH;99 (4 Supplement)():321-322
Aguas 2018	552	No outcomes reported	Infectivity of Chronic Malaria Infections and Its Consequences for Control and Elimination	2018. Clin Inf Dis;67(2):295-302
Ali 2017	522	Contextual Factors	Artemisinin combination therapy mass drug administration in a setting of low malaria endemicity: programmatic coverage and adherence during an observational study in Zanzibar	2017. Malaria journal;16(1):332
Archibald 1960	894	Intervention: not MDA	A preliminary report on the effect of diamino-diphenyl sulphone on malaria in northern Nigeria	1960. J Trop Med Hyg;63():25-7
Archibald 1960	895	Design: control group criteria not met	Field trials of mass administration of antimalarial drugs in Northern Nigeria	1960. ;262():44207
Archibald	896	Cross reference with outcomes; Linked to #895	The appearance of P. falciparum resistant to pyrimethamine in a	1960. West Afr Med

1960		(Design: control group criteria not met)	northern Nigerian village	J;9():21-5
Aregawi 2016	481	Setting: emergencies/epidemics	Impact of the Mass Drug Administration for malaria in response to the Ebola outbreak in Sierra Leone	2016. Malaria journal;15():480
Baukapur 1984	787	Setting: emergencies/epidemics	A focal outbreak of malaria in Valsad District, Gujarat State	1984. Journal of Communicable Diseases;16(4):268-272
Bertozzi-Villa 2017	120	Modelling data: ABSTRACT ONLY	Impact of human migration patterns on malaria elimination feasibility in the greater mekong subregion	2017. AJTMH;97 (5 Supplement 1)():522-523
Bloch 1982	786	Intervention: Insufficient information on drug administration;	Teachings of the Antimalarial Campaign in El Salvador, Central America	1982. R.I.I.M;11(2):119-124
Mason 1973	765	Intervention: Insufficient information on drug administration; linked ot Bloch 1982	A study of the epidemiology of malaria in a high-incidence coastal area of El Salvador, C. A	1973. Revista del Instituto de Investigaciones medicas;2(1):51-54, 55-57
Mason 1977	766	Intervention: No data on drug dose available: linked to Bloch 1982 (786)	Malaria field studies in a high-incidence coastal area of El Salvador, C.A	1977. Bulletin of the Pan American Health Organization;11(1):17-30
NaveReboll o 1973	763	Intervention: not enough data in main article for ITS, no control group; Insufficient information on drug administration Linked to Bloch 1982 (786)	Malaria in El Salvador. Control and eradication campaign analysis	1973. Revista del Instituto de Investigaciones medicas;2(1):31-39, 3-30
Boni 2019	174	No outcomes reported;	Antimalarial drug-resistance evolution during and after mass drug administration	2019. AJTMH;101 (5 Supplement)():510
Brady 2017	514	Modelling data;	Role of mass drug administration in elimination of Plasmodium falciparum malaria: a consensus modelling study	2017. The Lancet. Global health;5(7):e680-e687
Bretscher 2017	523	Modelling data;	Modelling the benefits of long-acting or transmission-blocking drugs for reducing Plasmodium falciparum transmission by case management or by mass treatment	2017. Malaria journal;16(1):341
Galatas 2020	635	Design: imbalance of interventions; blanket IRS began at same time as MDA	A multiphase program for malaria elimination in southern Mozambique (the Magude project): A before-after study	2020. PLoS medicine;17(8):e1003227
Galatas 2019	193	Duplicate; ASTMH abstract of publication #635 Galatas 2020	The magude project: Drastic reduction of malaria burden and sustained gains after a malaria elimination project in Southern Mozambique	2019. AJTMH;101 (5 Supplement)():417
Brew 2017	115	Duplicate; ABSTRACT; linked to Galatas 2020 (635)	The economic and educational impacts of a malaria elimination campaign in Mozambique	2017. AJTMH;97 (5 Supplement 1)():501
Cirera 2020	634	Contextual Factors; linked to 635, Galatas 2020	Moving towards malaria elimination in southern Mozambique: Cost and cost-effectiveness of mass drug administration combined with intensified malaria control	2020. PloS one;15(7):e0235631
Briet 2017	96	No outcomes reported;	Modelling to support the planning of malaria elimination in southern Palawan, the Philippines	2017. Trop Med Int Health;22 (Supplement 1)():55
Butler 1943	783	No outcomes reported;	Malaria Control Program on a South Pacific Base	1943. Naval Medical Bulletin;41(6):1603-12
Caceres	893	Cross reference with additional details; linked to	Eficacia de la cura radical masiva en la incidencia malÃ¡rica del	2004. Bol. malariol. salud

2004		Caceres 2005, 2008 (included)	Municipio Marino, Estado Sucre	ambient;():45-49
Caceres 2005	891	Cross reference with additional details; linked to Caceres 2008 (included)	Impacto de la Cura Radical Masiva sobre la incidencia malÃ¡rica del estado Sucre, Venezuela	2005. Bol. Malariol. Salud Amb;45():27-36
Camponovo 2019	615	Modelling data;	Mass campaigns combining antimalarial drugs and anti-infective vaccines as seasonal interventions for malaria control, elimination and prevention of resurgence: a modelling study	2019. BMC infectious diseases;19(1):920
Canet 1949	781	No outcomes reported	First Trials in Southern Indo-China of Mass Prophylaxis of Malaria with Nivaquine B (Resoqulne) and with Paludrine	1949. Bulletin de la Societe de Pathologie Exotique;42(44322):165-8
Canet 1949	844	Duplicate; 781 Canet 1949	First trials in souther Indo-China of mass prophylaxis of malaria with Nivaquine B (resoqulne) and with Paludrine.	1949. Bulletin de la Societe de Pathologie Exotique;42(44322):165-168
Cavalie 1961	890	Design: <2 areas/clusters per group	La campagne expeÌ�rimentale d'eÌ�radication du paludisme dans le Nord de la ReÌ�publique du Cameroun	1961. ;():
Celli 1903	859	Design: control group criteria not met	La malaria in Italia durante il 1902. Parte II: Profilassi della malaria. [Malaria in Italy in 1902. Part II: Prophylaxis of Malaria]	1903. Annali di Igiene Sperimentale;13():322-343
Celli 1914	843	No outcomes reported	[English title not available.]	1914. Ann. dâ€™Ingiene;24(2):177-243
Snowden 2006	799	Cross reference; linked to Celli 1914 (843)	Conquest of malaria: Italy, 1900-1962.	2006. ;():
Chang 2019	192	Design: control group criteria not met	Results of a pilot of targeted mass drug administration with sulfadoxine-pyrimethamine and primaquine as a component of a malaria elimination package in Haiti	2019. AJTMH;101 (5 Supplement)():417
Chang 2019	182	No outcomes reported	Accounting for human mobility in malaria elimination programs with heterogeneous travel data	2019. AJTMH;101 (5 Supplement)():189
Andrinopoulos 2016	78	Contextual Factors; Linked to Chang 2019 (192); control group criteria not met	Designing malaria elimination strategies to achieve high community uptake: Findings from a formative research study in the department of Grand Anse, Haiti	2016. AJTMH;95 (5 Supplement 1)():485
Druetz 2019	171	Cross reference with outcomes; linked to Chang 2019	Reduction in malaria prevalence in sentinel populations following introduction of a package of interventions for malaria elimination: Results from easy access group surveys in 2017 and 2018, Grande-Anse (Haiti)	2019. AJTMH;101 (5 Supplement)():523
Chaves 2020	712	No outcomes reported; Include for qualitative synthesis. No control group, vivax only	Malaria Elimination in Costa Rica: Changes in Treatment and Mass Drug Administration	2020. Microorganisms;8(7):
MarinRodriguez 2019	627	Linked to Chaves 2020	Parasite Removal for Malaria Elimination in Costa Rica	2019. Trends in parasitology;35(8):585-588
Cheah 2016	478	Contextual Factors;	Antimalarial mass drug administration: ethical considerations	2016. International health;8(4):235-8
Chen 1999	780	Intervention: not MDA; Tx not administered to entire	[A pilot study on malaria control by using a new strategy of	1999. Chung-Kuo Chi

		population	combining strengthening infection source treatment and health education in mountainous areas of Hainan province]	Sheng Chung Hsueh Yu Chi Sheng Chung Ping Tsa Chih Chinese Journal of Parasitology & Parasitic Diseases;17(1):44200
Chi 2017	669	Awaiting classification; This study does not yet appear to have been published.	Protocol of the Project of Malaria Elimination in the Plateaux Region of the Togolese Republic (Sino-Togolese Cooperation)	2017. http://www.who.int/trialsearch/Trial2.aspx?TrialID=ChiCTR-EON-17010697;():
Citron 2019	175	No outcomes reported; Abstract only	Quantifying malaria acquired during travel and its role in malaria elimination on Bioko Island	2019. AJTMH;101 (5 Supplement)():511
Ciuca 1937	840	Design: control group criteria not met	Experimental control of malaria with synthetic drugs.	1937. Archives Roumaines de Pathologie Experimentale et de Microbiologie;10(3):295-306
Clyde 1958	777	Intervention: exclude as targeted MDA (sent to emergency settings review)	Single Dose Pyrimethamine Treatment of Africans during a Malaria Epidemic in Tanganyika	1958. East African Medical Journal;35(1):23-9
Clyde 1961	778	Contextual Factors;	Malaria Control in Tanganyika under the German Administration. Part II. Mass Chcmoprophylaxis in Dar es Salaam	1961. East African Medical Journal;38(2):69-82
Clyde 1961	779	Cross reference with additional details; Linked to 778 Clyde 1961	Malaria Control in Tanganyika under the German Administration. Part I	1961. East African Medical Journal;38(1):27-42
Clyde 1961	838	Duplicate; Linked to 778 Clyde 1961	Malaria control of Tanganyika under the German Administration. II. Mass chemoprophylaxis in Dar es Salaam	1961. East Afr Med J;38():69-82
Dapeng 1996	774	Design: No pre-intervention data or control;	A successful control programme for falciparum malaria in Xinyang, China	1996. Trans R Soc Trop Med Hyg;90(2):100-2
Deng 2018	591	Design: control group criteria not met; not designed as ITS; no control group	Large-scale Artemisinin-Piperaquine Mass Drug Administration With or Without Primaquine Dramatically Reduces Malaria in a Highly Endemic Region of Africa	2018. Clin Inf Dis;67(11):1670-1676
Desowitz 1987	834	Intervention: not MDA;	Malaria in the Maprik area of the Sepik region, Papua New Guinea: 1957â€“1984	1987. Trans Royal Soc Trop Med Hyg;81(1):175-176
DeZulueta 1961	888	Design: control group criteria not met; Pf, no control, only 1 area	The results of the first year of a malaria eradication pilot project in Northern Kigezi (Uganda)	1961. East Afr Med J;38():44222
Diallo 1983	833	Intervention: not MDA; Treatment not administered to entire population	Clinical consequences of chloroquine prophylaxis and of its discontinuation in an hyperendemic malarial region	1983. Dakar Medical;28(1):43-65
Diallo 2016	70	Design: control group criteria not met	Targetting malaria hotspots in senegal: Results of a cluster-randomized trial	2016. AJTMH;95 (5 Supplement 1)():381
Diallo 2014	408	Cross reference with additional details; linked to Diallo 2016	A cluster-randomized trial of targeted control to eliminate malaria in central Senegal: Study design and acceptability of the interventions	2014. AJTMH;1)():198
Diallo 2015	32	Cross reference with outcomes; linked to Diallo 2016	A cluster-randomized trial of targeted control to eliminate malaria in central Senegal: Main results in year 2	2015. AJTMH;93 (4 Supplement)():81

Pactr 2013	276	Cross reference with additional details; trial registration; linked to Diallo 2016 (70)	A trial of targetted control to eliminate malaria in Central Senegal	2013. http://www.who.int/trialsearch/Trial2.aspx?TrialID=PACTR201310000575267;( ):
Tairou 2015	33	Contextual Factors; linked to Diallo 2016 (70)	The costs and cost-effectiveness of two spatially targeted, multi-component malaria elimination strategies: Results of a large three-arm cluster-randomized trial in rural Senegal	2015. AJTMH;93 (4 Supplement)():81
Dolenz 2013	378	No outcomes reported; This is an abstract, doesn't appear to be very relevant for modelling outcomes and has not been published.	Assessing cost optimized strategies for maintaining and extending the gains against malaria	2013. AJTMH;1)():262
Dupoux 1937	773	No outcomes reported;	Mass Prophylaxis of Malaria in Tunis	1937. Bull. Acad. Med.;118(35):368-372 pp.
Echodu 2018	149	Design: <2 areas/clusters per group; Excluded because each arm has a n=1	Mass drug administration combined with indoor residual spraying for accelerated reduction of malaria in a high transmission setting in northeastern Uganda: Preliminary results	2018. AJTMH;99 (4 Supplement)():580
Wanzira 2018	563	Contextual Factors; linked to Echodu 2018	Community facilitators and barriers to a successful implementation of mass drug administration and indoor residual spraying for malaria prevention in Uganda: a qualitative study	2018. Malaria journal;17(1):474
Elliott 2018	549	No outcomes reported; A modeling study that does not compare outcomes according to operational design considerations; linked to Echodu 2018	Medical and entomological malarial interventions, a comparison and synergy of two control measures using a Ross/Macdonald model variant and openmalaria simulation	2018. Mathematical biosciences;300():187-200
Elliott 2019	571	No outcomes reported; linked to Echodu 2018	Synergy and timing: a concurrent mass medical campaign predicted to augment indoor residual spraying for malaria	2019. Malaria journal;18(1):160
Mulebeke 2018	145	Duplicate; ASTMH abstract; linked to Echodu 2018	Implementing malaria mass drug administration: Experience from a high transmission setting in northeastern uganda	2018. AJTMH;99 (4 Supplement)():575
Mulebeke 2019	582	Contextual Factors; linked to Echodu 2018 (149)	Implementing population-based mass drug administration for malaria: experience from a high transmission setting in North Eastern Uganda	2019. Malaria journal;18(1):271
Escudie 1961	750	Design: <2 areas/clusters per group; See Escudie 1962	[Results of 2 years of antimalarial chemoprophylaxis in the rural African area in the pilot zone of Bobo Dioulasso (Haute Volta)]	1961. Med Trop (Mars);21(Special)():689-728
Escudie 1962	751	Cross reference with additional details linked to Escudie 1961	Results of mass antimalarial chemoprophylaxis with a combination of 4-aminoquinoline and 8-aminoquinoline under rural African conditions in the region of Bobo-Dioulasso (Upper Volta) 1960. Comparative study in a zone treated with DDT and outside this zone.	1962. Medecine Tropicale;22(2):268-305
Ricosse 1959	870	Design: <2 areas/clusters per group;	Results of pyrimethamine chemoprophylaxis in a pilot antimalarial prevention study in Boboâ€Dioulasso [Resultats d'une experimentation de chimioprophylaxie par la pyrimethamine dans la zone pilote de lutte antipaludique de Boboâ€Diolasso]	1959. Bulletin de la Societe de Pathologie Exotique;52():516â€35

Farinaud 1934	831	Intervention: not MDA	[English title not available] [Essai de prophylaxie rationelle du paludisme en milieu infantile a Tri-Cu (Tonkin)].	1934. Bulletin de la Societe de Pathologie Exotique;627(6):568-575
Farinaud 1958	898	Intervention: not MDA	Rapport sur les conditions d'organisation d'une campagne d'eradication due paludisme en Tunisie	1958. ;(EM/MAL/33):
Finda 2019	178	Irrelevant, no discussion of MDA	Perspectives of key stakeholders in Tanzania on alternative technologies for malaria elimination	2019. AJTMH;101 (5 Supplement)():124
Gao 2020	647	Modelling data;	Determinants of MDA impact and designing MDAs towards malaria elimination	2020. eLife;9():
Garbern 2018	134	Setting: emergencies/epidemics: https://academic.oup.com/ofid/article/6/7/ofz250/5498323	Effect of mass artesunate-amodioquine distribution on Ebola-related mortality in Sierra Leone	2018. AJTMH;99 (4 Supplement)():303
Garcia 2004	884	Cross reference with outcomes; no control, only 1 pre-intervention time point	Estado Sucre: El Ã©xito antimalÃ¡rico de Venezuela en el aÃ±o 2003	2004. BÃ³letin de MalariologÃa y Salud Ambiental;44(1):51-5
Garfield 1983	883	Imbalance of background interventions;	Changes in malaria incidence after mass drug administration in Nicaragua	1983. The Lancet;322(8348):500-503
Garfield 1986	882	Contextual Factors; linked to Garfield 1983 (883)	Health education and community participation in mass drug administration for malaria in Nicaragua	1986. Soc Sci Med;22(8):869-77
Gerardin 2015	245	Modelling data;	Mass campaigns with antimalarial drugs: a modelling comparison of artemether-lumefantrine and DHA-piperaquine with and without primaquine as tools for malaria control and elimination	2015. BMC infectious diseases;15():144
Gerardin 2016	462	Modelling data;	Optimal Population-Level Infection Detection Strategies for Malaria Control and Elimination in a Spatial Model of Malaria Transmission	2016. PLoS computational biology;12(1):e1004707
Gerardin 2018	545	Modelling data;	Impact of mass drug administration campaigns depends on interaction with seasonal human movement	2018. International health;10(4):252-257
Gomez Mendoza 1960	830	No outcomes reported	Observations on the programme for the employment of antimalarial drugs in the malaria eradication campaign in Venezuela.	1960. CNEP Boletin;4(2):74-81
Gomez Mendoza 1960	858	Duplicate; 830	Observations on the Programme for the Employment of Antimalarial Drugs in the Malaria Eradication Campaign in Venezuela [Informe del viaje efectuado para observar el programa de utilizaciÃ³n de drogas antipalÃºdicas en la campaÃ±a de erradicaciÃ³n del paludismo en la RepÃºblica de Venezuela.]	1960. CNEP Boletin;4():74-81
Gunther 1952	828	Intervention: not MDA	Proguanil and malaria among non-tolerant New Guinea natives.	1952. Trans Royal Soc Trop Med Hyg;46(2):185-200
Gupta 2020	716	Design: insufficient data points for ITS	Effect of mass dihydroartemisinin-piperaquine administration in southern Mozambique on the carriage of molecular markers of antimalarial resistance	2020. PloS one;15(10):e0240174
Henderson	827	Intervention: not tx dose; 1 area divided into 2 parts.	Prophylaxis of malaria in the Sudan, with special reference to the	1934. Trans Royal Soc Trop

1934		No clear data on prevalence pre-Treatment	use of plasmoquine	Med Hyg;28(2):157-164
Hii 1987	880	Design: control group criteria not met; MDA+ nets vs C: MDA. Does not meet design criteria but may have info on contextual factors	The influence of permethrin-impregnated bednets and mass drug administration on the incidence of Plasmodium falciparum malaria in children in Sabah, Malaysia	1987. Med Vet Entomol;1(4):397-407
Ho 1965	770	No outcomes reported	Studies on malaria in new China	1965. Chinese Medical Journal;84(8):491-497 pp.
Houel 1945	825	Intervention: not MDA	Chemoprophylaxis of malaria with monthly doses of choloroquine and amodiaquine.	1945. Bulletin de la Societe de Pathologie Exotique;47(2):254-260
Houel 1954	879	Intervention: not MDA	Treatment of Epidemic-Malaria with a Single Dose of Pyrimethamine	1954. Bulletin de la Societe de Pathologie Exotique;47(2):262-4
Hsiang 2013	233	Design: No pre-intervention data or control	Mass drug administration for the control and elimination of Plasmodium vivax malaria: an ecological study from Jiangsu province, China	2013. Malaria journal;12():383
Zhang 2014	690	Cross reference with outcomes; Linked Hsiang 2013 (233)	Preparation of malaria resurgence in China: case study of vivax malaria re-emergence and outbreak in Huang-Huai Plain in 2006	2014. Advances in parasitology;86():205-30
Huehne 1971	824	Design: control group criteria not met; uncontrolled. Only 1 site. Pf	Experience with an insecticide-drug combination and observations on suppressive chloroquine-pyrimethamine treatment	1971. J Trop Med Hyg;74(5):110-6
Husson 1906	897	Intervention: not MDA	Etiudes Epidemiologiques & Prophylactiques Sur Le Paludisme en Tunisie	1906. ;():
Jones 1954	878	Cross reference with outcomes; Linked to Jones 1958 (included)	Resistance of P. falciparum and P. malariae to Pyrimethamine (Daraprim) following Mass Treatment with this Drug. A Preliminary Note	1954. East African medical journal;31(2):47-9
Kaehler 2019	589	Contextual Factors;	The promise, problems and pitfalls of mass drug administration for malaria elimination: a qualitative study with scientists and policymakers	2019. International health;11(3):166-176
Kagaya 2019	642	Design: <2 areas/clusters per group	Malaria resurgence after significant reduction by mass drug administration on Ngodhe Island, Kenya	2019. Scientific reports;9(1):19060
Kagaya 2017	100	Duplicate; ASTMH abstract - duplicate of publication #642 (Kagaya 2019)	The impact of mass drug administration on submicroscopic malaria infection: A pilot study on ngodhe island in lake victoria, kenya	2017. AJTMH;97 (5 Supplement 1)():101
Idris 2016	485	No outcomes reported; linked to 642 (Kagaya 2019)	High and Heterogeneous Prevalence of Asymptomatic and Sub-microscopic Malaria Infections on Islands in Lake Victoria, Kenya	2016. Scientific reports;6():36958
Kaneko 2000	748	Imbalance of background interventions	Malaria eradication on islands	2000. Lancet;356(9241):1560-4
Kaneko 1994	876	Duplicate; linked to Kaneko 2000	Island malaria control in eastern Melanesia: 1) Malaria eliminated from a small island by 9-week mass drug administration and impregnated bednets	1994. J Japan. J. Parasitol.;43():358-370
Kaneko 2010	823	Contextual Factors; linked to Kaneko 2000	A community-directed strategy for sustainable malaria elimination on islands: short-term MDA integrated with ITNs and	2010. Acta Trop;114(3):177-83

			robust surveillance	
Kaneko 2014	407	Duplicate; linked to Kaneko 2000	Sustainable malaria elimination on aneityum Island, vanuatu, 1991-2014	2014. AJTMH;1)():197
Kaneko 2014	399	Cross reference with outcomes; Kaneko 2000	Community-directed malaria freedom on Aneityum Island, Vanuatu, 1991-2014	2014. Malaria Journal;1)():S25
Karl 2015	440	Modelling data;	Mathematical models for P. vivax elimination	2015. Trop Med Int Health;1)():105
Kasereka 2014	404	Setting: emergencies/epidemics;	Malaria case-finding and treatment strategies in an internally displaced persons (IDP) camp in the democratic republic of Congo	2014. AJTMH;1)():105
Khaing 2016	75	Awaiting classification; abstract only;	Evaluation of targeted mass treatment of malaria in tanintharyi region, myanmar: Preliminary results	2016. AJTMH;95 (5 Supplement 1)():401
Kligler 1931	874	Design: control group criteria not met;	Periodic Intermittent Treatment with Chinoplasmine as a Measure of Malaria Control in a Hyperendemic Area	1931. Riv Malariol;10(4):
Kobylinski 2020	652	Intervention: not MDA;	Safety, Pharmacokinetics, and Mosquito-Lethal Effects of Ivermectin in Combination With Dihydroartemisinin-Piperaquine and Primaquine in Healthy Adult Thai Subjects	2020. Clinical pharmacology and therapeutics;107(5):1221-1230
Kuehne 2015	439	Setting: emergencies/epidemics; abstract	Malaria prevalence decreased following mass drug administration of malaria chemoprevention during the Ebola outbreak, Monrovia, Liberia, 2014	2015. Trop Med Int Health;1)():43-44
Kuehne 2016	480	Setting: emergencies/epidemics	Impact and Lessons Learned from Mass Drug Administrations of Malaria Chemoprevention during the Ebola Outbreak in Monrovia, Liberia, 2014	2016. PloS one;11(8):e0161311
Kyaw 2019	176	Contextual Factors; abstract only, no data at all included here	Costing mass drug administration with different targeting strategies	2019. AJTMH;101 (5 Supplement)():123
Landier 2018	607	Design: control group criteria not met	Effect of generalised access to early diagnosis and treatment and targeted mass drug administration on Plasmodium falciparum malaria in Eastern Myanmar: an observational study of a regional elimination programme	2018. Lancet (London, England);391(10133):1916-1926
Levenson 1943	819	Design: No pre-intervention data or control	Experiences in the control of a malarial focus in the north (Arehangel RÃ©gion) by mass chemoprophylaxis and systemic treatment of malaria patients (Russian).	1943. Meditsinkaya Parazitologiya i Parazitarnya Bolezni;12():23-38
Liu 1986	818	Intervention: not tx dose; mixed curative and prophylactic dose;	INTEGRATED APPROACH IN MALARIA CONTROL INCLUDING ENVIRONMENTAL MANAGEMENT TO REDUCE MAN-MOSQUITO CONTACT AND REDUCTION OF INFECTION SOURCE IN HUANGHUAI PLAIN	1986. CHINESE JOURNAL OF PARASITOLOGY AND PARASITIC DISEASES;4(4):246-250
Lwin 2015	449	Design: control group criteria not met;	Elimination of Plasmodium falciparum in an area of multi-drug resistance	2015. Malaria journal;14():319
Lysenko 1960	817	No outcomes reported;	Use of quinocide in treatment and prophylaxis of vivax malaria	1960. Bull World Health Organ;22(6):641-62
Muhlens	810	Intervention: not MDA;	Report of a malaria expedition to Jerusalem.	1913. Zentrablatt fur

1913				Backteriologie, Parasitenkunde, Infektionskrankheiten und Hygiene;69(44198):41-85
Sok 2016	71	Intervention: not MDA	Comparison of mass drug administration vs. mass screening and treatment high-risk, military mobile populations to support malaria elimination in Cambodia	2016. AJTMH;95 (5 Supplement 1)():381
Wojnarski 2016	86	Cross reference with outcomes; MAIN: Sok 2016 (71)	Primaquine safety in G6PD-deficient military cohort in cambodia using the lower-dose, extended course regimen as part of mass drug administration for malaria elimination	2016. AJTMH;95 (5 Supplement 1)():281
	18	Cross reference with additional details; Linked to Sok 2016 (71)	Malaria Elimination Pilot Study in Military Forces in Cambodia	. ;():
Manning 2018	558	Cross reference with additional details; linked to Sok 2016	Cluster-randomized trial of monthly malaria prophylaxis versus focused screening and treatment: a study protocol to define malaria elimination strategies in Cambodia	2018. Trials;19(1):558
Manning 2018	668	Duplicate 558	Cluster-randomized trial of monthly malaria prophylaxis versus focused screening and treatment: a study protocol to define malaria elimination strategies in Cambodia 11 Medical and Health Sciences 1117 Public Health and Health Services	2018. Trials;19(1) (no pagination):
Marasinghe 2020	205	Intervention: not MDA;	Mass radical treatment of a group of foreign workers to mitigate the risk of re-establishment of malaria in Sri Lanka	2020. Malaria Journal;19 (1) (no pagination)(346):
Maude 2012	215	Modelling data; Kim Lindblade (2021-02-19 23:24:39)(Select): This is linked to Song 2010 #863, which is the main paper.; Monica Shah (2021-02-15 10:04:15)(Select): Update: this study is not linked to others. Screen this as an independent study;	Optimising strategies for Plasmodium falciparum malaria elimination in Cambodia: primaquine, mass drug administration and artemisinin resistance	2012. PloS one;7(5):e37166
Maude 2014	685	Modelling data;	The diminishing returns of atovaquone-proguanil for elimination of Plasmodium falciparum malaria: modelling mass drug administration and treatment	2014. Malaria journal;13():380
Maude 2016	65	No outcomes reported;	Mathematical modelling of tafenoquine for plasmodium falciparum malaria elimination	2016. AJTMH;95 (5 Supplement 1)():305
Mendez Galvan 1984	764	Intervention: can't determine when rounds occured. 3 rounds, monthly, uncontrolled study for pv	Evaluation of alternative scheme of treatment for malaria control	1984. Salud Publica de Mexico;26(6):561-572
Mendez Galvan 1984	813	Duplicate: 764	Evaluuation of alternative scheme of treatment for malaria control.	1984. Salud Publica de Mexico;26(6):561-572
Merle 1955	812	Intervention: not MDA: Treatment not administered to entire population.	[English title not available] [Problemas actuales del control y erradicacion de la malaria en America Latina].	1955. Bulletin de la Societe de Pathologie Exotique;48(2):242-269
Millar 2020	733	No outcomes reported;	To screen or not to screen: an interactive framework for comparing costs of mass malaria treatment interventions	2020. BMC medicine;18(1):149

Millat-Martinez 2018	557	Intervention: not MDA	Electrocardiographic Safety of Repeated Monthly Dihydroartemisinin-Piperaquine as a Candidate for Mass Drug Administration	2018. Antimicrobial agents and chemotherapy;62(12):
Mosha 2013	229	Intervention: not MDA;	Epidemiology of subpatent Plasmodium falciparum infection: implications for detection of hotspots with imperfect diagnostics	2013. Malaria journal;12():221
Murta 2019	576	Contextual Factors;	Misperceptions of patients and health workers regarding malaria elimination in the Brazilian Amazon: a qualitative study	2019. Malaria journal;18(1):223
Najera 1973	746	Imbalance of background interventions;	Mass drug administration and DDT indoor-spraying as antimalarial measures in the norther savanna of Nigeria	1973. World Health Organization;73(817):12420
Nankabirwa 2019	185	Modelling data;	Quantifying the potential impact of mass drug administration on the parasite reservoir in an area of declining malaria transmission in Uganda	2019. AJTMH;101 (5 Supplement)():316-317
Nct 2016	274	Registration for Morris 2018 (562) which was included	Effectiveness of Mass Drug Administration for Reducing Seasonal Malaria Transmission in Zanzibar	2016. https://clinicaltrials.gov/show/NCT02721186;():
Nct 2018	267	Intervention: not MDA; Protocol only. MDA Ivermectin + DP, no DP alone arm: https://pubmed.ncbi.nlm.nih.gov/33211022/;	Mass Drug Administration of Ivermectin and Dihydroartemisinin-piperaquine as an Additional Intervention for Malaria Elimination	2018. https://clinicaltrials.gov/show/NCT03576313;():
Nikolov 2015	447	No outcomes reported; Abstract only	Modeling the effectiveness of population-level malaria infection detection strategies for optimal campaign scoping	2015. AJTMH;93 (4 Supplement)():293
Norman 1952	762	No outcomes reported;	An Investigation of the Failure of Proguanil Prophylaxis	1952. Trans Royal Soc Trop Med Hyg;46(6):653-5
Nosten 2016	489	No outcomes reported; perspective piece;	[Elimination in South-East Asia? The role of antimalarial drugs]	2016. Elimination du paludisme en Asie du Sud-Est? Moyens medicamenteux.;200(3):467-6
Ossi 1967	761	Contextual Factors; Uncontrolled before and after for pv, but pre-intervention and post-intervention includes cases (no denominators) in different populations. Insufficient info on outcomes to include.	An epidemic in the life of a malaria eradication programme	1967. Bulletin of Endemic Diseases;9(44200):44334
Ossi 1967	806	Duplicate 761	An epidemic in the life of malaria eradication programme.	1967. Bulletin of Endemic Diseases;9():44334
Pemberton-Ross 2016	56	No outcomes reported; ASTMH abstract	Reactive case detection for malaria elimination	2016. AJTMH;95 (5 Supplement 1)():283
Pemberton-Ross 2017	528	Modelling data;	A stochastic model for the probability of malaria extinction by mass drug administration	2017. Malaria journal;16(1):376
Pikul 1934	760	Intervention: not MDA- this is MTaT	Experiment on the Prophylactic Use of Plasmocide in Daghestan with Observations on the Mosquito Infection Rate	1934. Meditsinskaya Parazitologiya i Parazitarnye Bolezni;3(4):322-329 pp.

Pikul 1934	805	Duplicate;	Experiment on the prohylactic use of plasmocide in Daghestan with observations on the mosquito infection rate.	1934. Meditsinkaya Parazitologiya i Parazitarnya Bolezni;3(4):322-329
Porter 2016	87	Duplicate;	Assessing associations between recent travel and malaria parasite prevalence during a mass drug administration campaign in southern zambia	2016. AJTMH;95 (5 Supplement 1)():282
Roberts 1956	745	Design: <2 areas/clusters per group;	Pyrimethamine (Daraprim) in the control of epidemic malaria	1956. J Trop Med Hyg;59(9):201-8
Robinson 2015	600	No outcomes reported; targeted MDA (to children)	Strategies for understanding and reducing the Plasmodium vivax and Plasmodium ovale hypnozoite reservoir in Papua New Guinean children: a randomised placebo-controlled trial and mathematical model	2015. PLoS medicine;12(10):e1001891
Roy 2013	395	Intervention: not tx dose; Included in Mass relapse prevention review	The Potential Elimination of Plasmodium vivax Malaria by Relapse Treatment: Insights from a Transmission Model and Surveillance Data from NW India	2013. PLoS Neglected Tropical Diseases;7 (1) (no pagination)(e1979):
Runge 2020	625	Modelling data;	Simulating the council-specific impact of anti-malaria interventions: A tool to support malaria strategic planning in Tanzania	2020. PloS one;15(2):e0228469
Saarinen 1987	804	Intervention: not MDA;	Mass proguanil prophylaxis	1987. The Lancet;1(8539):985-986
Selvaraj 2019	613	Intervention: not MDA;	Reducing malaria burden and accelerating elimination with long-lasting systemic insecticides: a modelling study of three potential use cases	2019. Malaria journal;18(1):307
Sergent 1913	758	No outcomes reported	[English title not available]	1913. Ann. Inst. Pasteur;27(5):373-390
Sergent 1913	801	Duplicate 758	[Etudes epidemiologiques et prophylactiques du paludisme: neuvieme et dixieme campagnes en Algerie, en 1910 et 1911]	1913. Annales de lâ€™Institut Pasteur;27(5):373-390
Silal 2014	411	Duplicate 681	Towards malaria elimination in Mpumalanga, South Africa: A metapopulation modeling approach	2014. AJTMH;1)():278
Silal 2014	681	Modelling data; LINKED: 411-dup681	Towards malaria elimination in Mpumalanga, South Africa: a population-level mathematical modelling approach	2014. Malaria journal;13():297
Silal 2015	43	No outcomes reported; Abstract only;	Modelling mass drug administration in malariaendemic countries in the presence of imported infections	2015. AJTMH;93 (4 Supplement)():289
Silal 2015	255	Intervention: not MDA;	Predicting the impact of border control on malaria transmission: a simulated focal screen and treat campaign	2015. Malaria journal;14():268
Silal 2015	457	No outcomes reported;	Hitting a Moving Target: A Model for Malaria Elimination in the Presence of Population Movement	2015. PloS one;10(12):e0144990
Silal 2017	106	Contextual Factors; abstract only;	Costing malaria elimination in the Asia-pacific	2017. AJTMH;97 (5 Supplement 1)():332-333
Simeons	865	Cross reference with outcomes; Simeons 1936	Follow-Up of a Mass Treatment with Injectable Atebrin	1938. Ind Med

1938		(included)		Gaz;73(12):713-715
Singh 1953	742	Intervention: not tx dose;	Suppressive treatment with amodiaquin	1953. Indian J Malariol;7(1):27-31
Singh 1968	800	No outcomes reported	Epidemiological study of focal outbreak of malaria in consolidation phase area and evaluation of remedial measures in Uttar Pradesh (India).	1968. Bulletin of the Indian Society for Malaria and Other Communicable Diseases;5():207-220
Slater 2014	679	Modelling data;	The potential impact of adding ivermectin to a mass treatment intervention to reduce malaria transmission: a modelling study	2014. J Inf Dis;210(12):1972-80
Smith 2019	621	No outcomes reported;	Resurgence of malaria infection after mass treatment: a simulation study	2019. Malaria journal;18(1):409
Stuckey 2014	418	Duplicate;	Investigating operational strategies for antimalarial drug administration in Zambia's Southern province: A simulation study	2014. AJTMH;1)():461
Toyb 2016	329	Design: control group criteria not met	Le paludisme dans l'Archipel des Comores : Ã©tat des lieux en 2015 aprÃ¨s quinze annÃ©es de lutte	2016. Bull Soc Pathol Exot;109(2):107-13
VanDijk 1961	862	Intervention: not tx dose;	Mass treatment of malaria with chloroquine. Results of a trial in Inanwatan	1961. Tropical geographical medicine;13(4):351-6
Walker 2015	25	Duplicate #475 Walker;	Estimating the most resource-efficient malaria intervention packages and spatial scales to achieve elimination across Africa	2015. AJTMH;93 (4 Supplement)():476-477
Walker 2015	46	Duplicate;	Estimated increase in malaria morbidity and mortality in EBOLA-affected countries due to decreased healthcare capacity and the potential impact of mitigation strategies	2015. AJTMH;93 (4 Supplement)():292
Walker 2015	247	Setting: emergencies/epidemics;	Malaria morbidity and mortality in Ebola-affected countries caused by decreased health-care capacity, and the potential effect of mitigation strategies: a modelling analysis	2015. The Lancet. Infectious diseases;15(7):825-32
Walker 2016	475	Modelling data;	Estimating the most efficient allocation of interventions to achieve reductions in Plasmodium falciparum malaria burden and transmission in Africa: a modelling study	2016. The Lancet. Global health;4(7):e474-84
Wanzira 2018	131	Duplicate; ASTMH Abstract ;	Community facilitators and barriers to a successful implementation of mass drug administration and indoor residual spraying for malaria prevention in Uganda	2018. AJTMH;99 (4 Supplement)():349
Yukich 2016	76	Duplicate;	Cost-effectiveness of focal mass drug administration and mass drug administration with dihydroartemisinin-piperaquine for malaria prevention in Southern Province, Zambia: Results of a community randomized control trial	2016. AJTMH;95 (5 Supplement 1)():8
Zulueta 1964	860	Design: control group criteria not met; MDA given concomitantly with DDT spray rounds, no control group (Pf)	A Malaria Eradication Experiment in the Highlands of Kigezi (Uganda)	1964. East Afr Med J;41():102-20
Lakshmana charyulu	767	Intervention: not MDA	Control of Malaria Epidemics in a River Valley project	. ;():312-322
White 1937	793	Intervention: not MDA; Abstract	Anti-gametocyte treatment combined with anti-larval malaria	1937. Records of the

			control. Part II.	Malaria Survey of India;7(4):221-231
Xu 2022	920	Contextual Factors;	Mass drug administration in response to vivax malaria resurgence in Anhui Province of Huanghuai Plain, China	2022. Advances in parasitology;116():115-152
Li 2021	986	Contextual Factors;	Seven decades towards malaria elimination in Yunnan, China	2021. Malaria journal;20(1):147
Fehr 2021	965	Contextual Factors;	From informed consent to adherence: factors influencing involvement in mass drug administration with ivermectin for malaria elimination in The Gambia	2021. Malaria journal;20(1):198
Keys 2021	988	Contextual Factors;	Rapid ethnographic assessment for potential anti-malarial mass drug administration in an outbreak area of Santo Domingo, Dominican Republic	2021. Malaria journal;20(1):76
vanBeek 2021	953	Modelling data;	Model-based assessment of the safety of community interventions with primaquine in sub-Saharan Africa	2021. Parasites & vectors;14(1):524
Aung 2021	949	Contextual Factors;	The acceptability of targeted mass treatment with primaquine for local elimination of vivax malaria in a northern Myanmar township: a mixed-methods study	2021. Parasites & vectors;14(1):549
Cheng 2021	979	Contextual Factors;	A systematic review of factors influencing participation in two types of malaria prevention intervention in Southeast Asia	2021. Malaria journal;20(1):195
Fehr 2021	951	Contextual Factors;	The role of social cohesion in the implementation and coverage of a mass drug administration trial for malaria control in the Gambia: An in-depth comparison of two intervention villages	2021. Social science & medicine (1982);291():114487
Tian 2022	900	Modelling data;	Malaria elimination on Hainan Island despite climate change	2022. Communications medicine;2():12
Galatas 2021	983	Contextual Factors;	Community acceptability to antimalarial mass drug administrations in Magude district, Southern Mozambique: A mixed methods study	2021. PloS one;16(3):e0249080
Thomas 2021	975	Imbalance of background interventions\	The short-term impact of a malaria elimination initiative in Southern Mozambique: Application of the synthetic control method to routine surveillance data	2021. Health economics;30(9):2168-2184
Nadia 2022	966	No outcomes reported;	Historical experiences on mass drug administration for malaria control and elimination, its challenges and China's experience: a narrative review	2022. Acta tropica;225():106209
Nct 2021	1078	Awaiting classification;	Mass Drug Administration of Dihydroartemisinin-piperaquine + Single Low-dose Primaquine to Accelerate Toward Elimination Activities	2021. https://clinicaltrials.gov/show/NCT04864444;():
Liu 2022	923	Design: control group criteria not met; no control, impossible to separate effect of MDA from other interventions;	Malaria from hyperendemicity to elimination along international borders in Yunnan, China during 2003-2020: a case study	2022. Infectious diseases of poverty;11(1):51
Li 2021	924	Design: <2 areas/clusters per group;	Mass Drug Administration With Artemisinin-Piperaquine for the Elimination of Residual Foci of Malaria in Sao Tome Island	2021. Frontiers in medicine;8():617195

Liu 2020	1039	No outcomes reported	Successful case studies on malaria elimination with multi-province cooperation in China	2020. American Journal of Tropical Medicine and Hygiene;103(5 SUPPL)():221
Wu 2020	1002	No outcomes reported;	Sero-epidemiological evaluation of malaria transmission in The Gambia before and after mass drug administration	2020. BMC medicine;18(1):331
Kyaw 2021	977	Modelling data	Estimating the programmatic cost of targeted mass drug administration for malaria in Myanmar	2021. BMC public health;21(1):826
Dabira 2022	932	Imbalance of background interventions\	Mass drug administration of ivermectin and dihydroartemisinin-piperaquine against malaria in settings with high coverage of standard control interventions: a cluster-randomised controlled trial in The Gambia	2022. The Lancet. Infectious diseases;22(4):519-528
Druetz 2022	909	Design: No pre-intervention data or control;	Etramp5 as a useful serological marker in children to assess the immediate effects of mass drug campaigns for malaria	2022. BMC infectious diseases;22(1):643
Druetz 2022	919	Imbalance of background interventions; Kim Lindblade (2022-08-16 02:41:13)(Select): PRE-POST STUDY WITH NON-RANDOMIZED CONTROLS; IRS IMPLEMENTED AT SAME TIME AS MDA;	The Immediate Effects of a Combined Mass Drug Administration and Indoor Residual Spraying Campaign to Accelerate Progress Toward Malaria Elimination in Grande-Anse, Haiti	2022. The Journal of infectious diseases;225(9):1611-1620
Searle 2020	1040	Irrelevant, no discussion of MDA;	Coverage, use, and impacts of plasmodium falciparum malaria prevention and control measures in rural sussundenga, Mozambique	2020. American Journal of Tropical Medicine and Hygiene;103(5 SUPPL)():343

Notes: MDA = mass drug administration

In order to ensure that each population was captured only once, when there were multiple publications from a single study, only one paper was included, however, the relevant data from excluded papers was still captured. This is indicated as cross reference with outcomes. Studies which contributed to the contextual factor and modelling synthesis are highlighted in blue and yellow, respectively.

Supplementary table 3. Coverage of vector control co-interventions

Study (location, years of study)	Co-intervention	IRS Coverage	ITN/LLIN coverage
**Cluster randomized trials**			
von Seidlein 2003[35] (Gambia, 1999)	MDA only		
Shekalaghe 2011[23] (Zanzibar, 2008)	ITN		Reported ITN use 25.1% to 36.1% (covers both intervention and comparison arms)
Morris 2018[22] (Zanzibar, 2016-2017)	ITN and IRS	Single round in March 2016 with pirimiphos methyl; 85% of households sprayed at baseline	Universal distribution campaign in 2015-2016; self-reported ITN use among all ages 71% at baseline
von Seidlein 2019[28] (Viet Nam, 2013-2014)	LLINs		LLIN use reported by 81.5% overall; 84.4% in control and 78.5% in intervention arms
von Seidlein 2019[28] (Myanmar 2013-2014)	LLINs		LLIN use reported by 81.5% overall; 84.4% in control and 78.5% in intervention arms
von Seidlein 2019[28] (Laos 2016-2017)	LLINs		LLIN use reported by 81.5% overall; 84.4% in control and 78.5% in intervention arms
von Seidlein 2019[28] (Cambodia 2014-2016)	LLINs		LLIN use reported by 81.5% overall; 84.4% in control and 78.5% in intervention arms
Eisele 2020A[21] (Zambia, 2014-2017) (Low transmission)	LLINs and IRS	6.9% in the MDA arm, 16.9% in control at baseline (2014); 42.2% in MDA and 47.8% in control by 2016	70.3% in MDA and 75.3% in control at baseline (2014) and 77.2% in MDA and 78.8% by 2016
Eisele 2020B[21] (Zambia, 2014-2017) (High transmission)	LLINs and IRS	6.9% in the MDA arm, 16.9% in control at baseline (2014); 42.2% in MDA and 47.8% in control by 2017	70.3% in MDA and 75.3% in control at baseline (2014) and 77.2% in MDA and 78.8% by 2017
McLean 2021[27] (Myanmar, 2014 to 2017)	LLIN		In all clusters, CHWs distributed LLINs (one per two people in each household)
**Non-randomized studies**			
Simeons 1938 [34] (India, 1935)	Oiling for Larval Control		

Jones 1958 [32] (Kenya, 1952-1953)	MDA only		
Gabaldon 1959[31] (Venezuela, 1956-1957)	IRS	Coverage not specified	
Schneider 1961[26] (Burkina Faso, 1960-1961)	Group 1: MDA only. Group 2: IRS	Interventon group 2 only: Co-intervention with IRS using DDT once a year in May 1960, coverage not specified	
Comer 1971[30] (Panama, 1965-1968)	MDA only		
Paik 1974A[33] (Solomon Islands, 1972)	IRS	"Total complete coverage" every 6 months	
Paik 1974B[33] (Solomon Islands, 1972-73)	MDA only		
Molineaux 1980[25] (Nigeria, 1970-1976)	IRS	~99% coverage with IRS using propoxur 3-4 rounds per year in both intervention and comparison arms	
Cáceres Garcia 2008[29] (Venezuela, 2002-2007)	MDA only		

Supplementary table 4. GRADE Summary of Findings Tables

Outcomes	Studies and participants	Rate ratio (95% CI)	Anticipated absolute effects* (95% CI) Risk with no MDA	Risk with MDA	Certainty
Pf Mod-high transmission					
AEs	1 RCT 90 participants	OR 3.25 (0.68 to 15.53)	133 per 1,000 person-years	333 per 1,000 (95 to 705)	Very low [a,d]
Pf low-very low transmission					
SAEs, 0-3 months post MDA	1 RCT 6911 participants	OR 3.61 (0.43 to 30.03)	0 per 1,000 person-years	1 per 1,000 (0 to 11)	Moderate [d]
SAEs 4-12 months post MDA	1 RCT 6911 participants	OR 1.47 (0.68 to 3.20)	3 per 1,000 person-years	5 per 1,000 (2 to 11)	Moderate [d]
Vomiting	1 RCT 703 participants	OR 0.54 (0.19 to 1.54)	43 per 1,000 person-years	24 per 1,000 (8 to 65)	Moderate [d]
Drug resistance markers (PfKelch13) among Pf positive individuals 1-3 months post MDA	1 RCT 63 participants	RR 0.82 (0.45 to 1.51)	608 per 1,000 person-years	498 per 1,000 (274 to 918)	Very low [a,d,k]
Drug resistance markers (PfKelch13) among all samples 1-3 months post MDA	1 RCT 1232 participants	RR 0.13 (0.05 to 0.30)	64 per 1,000 person-years	8 per 1,000 (3 to 19)	Low [a,k]
Drug resistance markers (PfKelch13) among Pf positive individuals 4-12 months post MDA	1 RCT 75 participants	RR 1.16 (0.83 to 1.61)	610 per 1,000 person-years	707 per 1,000 (506 to 982)	Very low [a,d,k]
Drug resistance markers (PfKelch13) among all samples 4-12 months post MDA	1 RCT 2595 participants	RR 0.49 (0.28 to 0.85)	29 per 1,000 person-years	14 per 1,000 (8 to 24)	Low [a,k]
Drug resistance markers (PfKelch13) among Pf positive individuals 12-24 months post MDA	1 RCT 78 participants	RR 1.07 (0.82 to 1.40)	714 per 1,000 person-years	764 per 1,000 (586 to 1,000)	Very low [a,d,k]
Drug resistance markers (PfKelch13) among all samples 2-24 months post MDA	1 RCT 2990 participants	RR 0.66 (0.40 to 1.11)	25 per 1,000 person-years	17 per 1,000 (10 to 28)	Low [a,k]
Pv					
SAEs, 0-3 months post MDA	1 RCT 6911 participants	OR 3.61 (0.43 to 30.03)	0 per 1,000 person-years	1 per 1,000 (0 to 11)	Moderate [h]
SAEs, 4-12 months post MDA	1 RCT 6911 participants	OR 1.47 (0.68 to 3.20)	3 per 1,000 person-years	5 per 1,000 (2 to 11)	Moderate [h]

Supplementary Figure 1a. Risk of bias for included studies; Randomized controlled trials

	Domain 1a.	Domain 1b.	Domain 2.	Domain 3.Parasitemia Prevalence	Domain 3. Parasitemia Incidence	Domain 3. Incidence of Confirmed Malaria Illness	Domain 4: Parasitemia Prevalence	Domain 4: Parasitemia Incidence	Domain 4: Incidence of Confirmed Malaria Illness	Domain 5.	OVERALL: Parasitemia Prevalence	OVERALL: Parasitemia Incidence	OVERALL: Incidence of Confirmed Malaria Illness
Cáceres Garcia 2008 Venezuela	+		+			−			−	−			−
Comer 1971 Panama	−		+	+			+			−	−		
Eisele 2020 Zambia A	+	+	+	+	+	+	+	+	?	+	+	+	?
Eisele 2020 Zambia B	+	+	+	+	+	+	+	+	?	+	+	+	?
Gabaldon 1959 Venezuela	?		?		+			+		−		−	
Jones 1954 Kenya	?		+	+			+			−	−		
McLean 2021 Myanmar	+	+	?	−			+			+	−		
Molineaux 1980 Nigeria	+		+	+			+			?	?		
Morris 2018 Zanzibar	?	+	+	+		+	+		?	+	?		?
Paik 1974 Solomon Islands A	+		+	?	?		+	+		−	−	−	
Paik 1974 Solomon Islands B	+		+		?			−		−		−	
Schneider 1961 Burkina Faso	?		+	+			+			?	?		
Shekalaghe 2011 Tanzania	+	+	+	+		+	+		+	+	+		+
Simeons 1938 India	+		+			−			−	−			−
von Seidlein 2003 Gambia	+	+	+	+	−		+	+		+	+	−	
von Seidlein 2019 Cambodia	?	+	+	+		+	+		?	+	?		?
von Seidlein 2019 Laos	?	+	+	+			+			+	?		
von Seidlein 2019 Myanmar	?	+	+	+		+	+		?	+	?		?
von Seidlein 2019 Viet Nam	+	+	?	+			+			+	?		

Randomized studies: ROB2 tool: Domain 1a. Risk of bias arising from randomization; Domain 1b. Risk of bias from timing of identification/ recruitment; Domain 2. Risk of bias due to deviations from intended intervention; Domain 3. Missing outcome data; Domain 4. Risk of bias in measurement of the outcome; Domain 5. Risk of bias in selection of reported result.

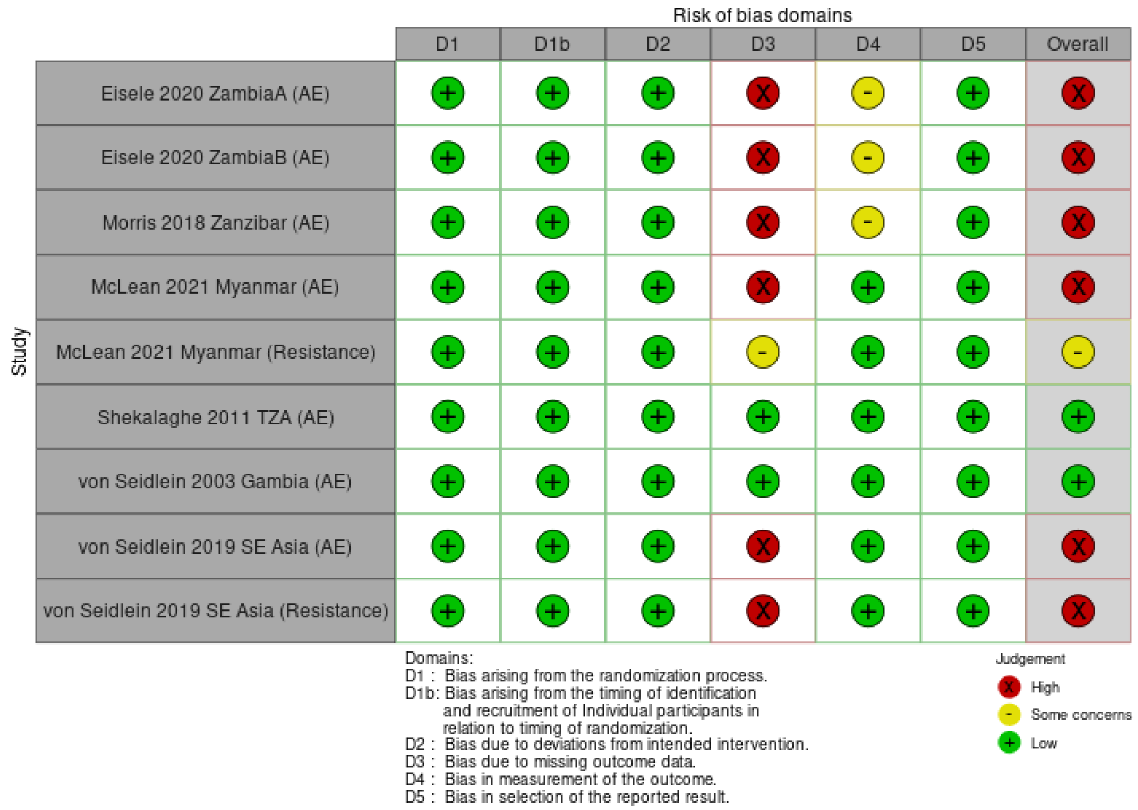

Supplementary Figure 1b. Risk of bias for included studies; Non-randomized trials

Non-randomized studies: Domain 1. Failure to develop and apply appropriate eligibility criteria (inclusion of control population); Domain 1b. Not applicable; Domain 2. Flawed measurement of exposure; Domain 3. Incomplete follow-up; Domain 4. Flawed measurement of outcome (outcome-level); Domain 5. Failure to adequately control for confounding.

Supplementary Figure 2. Prevalence of *P. falciparum* parasitemia in moderate- high transmission settings, Nonrandomized Trials

Study or Subgroup	MDA Events	MDA Total	Control Events	Control Total	Weight	Risk Ratio M-H, Fixed, 95% CI	Year
**2.1.1 < 1 Month**							
**Subtotal (95% CI)**		**0**		**0**		**Not estimable**	
Total events	0		0				
Heterogeneity: Not applicable							
Test for overall effect: Not applicable							
**2.1.2 1-3 Months**							
Schneider 1961 Burkina Faso	286	466	386	534	100.0%	0.85 [0.78, 0.93]	
**Subtotal (95% CI)**		**466**		**534**	**100.0%**	**0.85 [0.78, 0.93]**	
Total events	286		386				
Heterogeneity: Not applicable							
Test for overall effect: Z = 3.60 (P = 0.0003)							
**2.1.3 4-12 Months**							
Molineaux 1980 Nigeria (1)	523	2071	453	1083	100.0%	0.60 [0.55, 0.67]	1975
**Subtotal (95% CI)**		**2071**		**1083**	**100.0%**	**0.60 [0.55, 0.67]**	
Total events	523		453				
Heterogeneity: Not applicable							
Test for overall effect: Z = 9.69 (P < 0.00001)							
**2.1.4 12-24 Months**							
Molineaux 1980 Nigeria (2)	705	2138	484	1123	100.0%	0.77 [0.70, 0.84]	1975
**Subtotal (95% CI)**		**2138**		**1123**	**100.0%**	**0.77 [0.70, 0.84]**	
Total events	705		484				
Heterogeneity: Not applicable							
Test for overall effect: Z = 5.81 (P < 0.00001)							

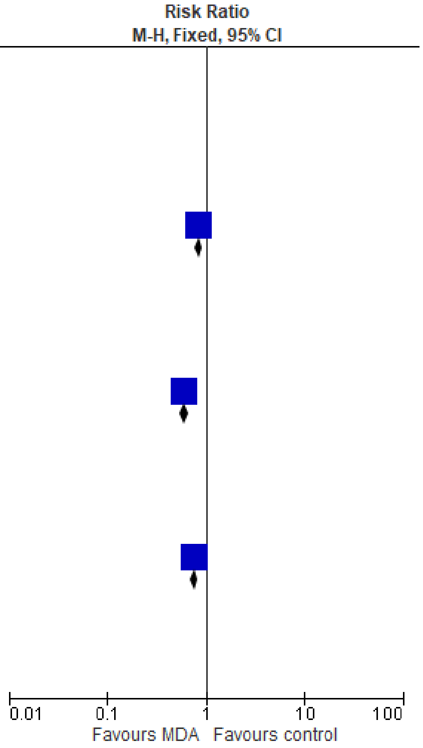

Test for subgroup differences: Chi² = 24.88, df = 2 (P < 0.00001), I² = 92.0%

Footnotes

(1) Molineaux 1980 NGA: MDA (SP every 2 weeks during the wet season and 10 weeks during the dry season) + IRS vs. no intervention

(2) Molineaux 1980 NGA: MDA (SP every 2 weeks during the wet season and 10 weeks during the dry season) + IRS vs. no intervention

Supplementary Figure 3. Incidence of clinical *P. falciparum* malaria in moderate- high transmission settings, cRCTs

Study or Subgroup	log[Rate Ratio]	SE	Weight	Rate Ratio IV, Fixed, 95% CI	Year
**3.1.1 MOD/HIGH 1-3 Mos. Post MDA**					
Eisele 2020 Zambia B (1)	-0.9	1.217	100.0%	0.41 [0.04, 4.42]	
**Subtotal (95% CI)**			**100.0%**	**0.41 [0.04, 4.42]**	
Heterogeneity: Not applicable					
Test for overall effect: Z = 0.74 (P = 0.46)					
**3.1.2 MOD/HIGH 4-12 Mos. Post MDA**					
**Subtotal (95% CI)**				**Not estimable**	
Heterogeneity: Not applicable					
Test for overall effect: Not applicable					
**3.1.3 MOD/HIGH 12-24 Mos. Post MDA**					
**Subtotal (95% CI)**				**Not estimable**	
Heterogeneity: Not applicable					
Test for overall effect: Not applicable					
**Total (95% CI)**			**100.0%**	**0.41 [0.04, 4.42]**	
Heterogeneity: Not applicable					
Test for overall effect: Z = 0.74 (P = 0.46)					
Test for subgroup differences: Not applicable					

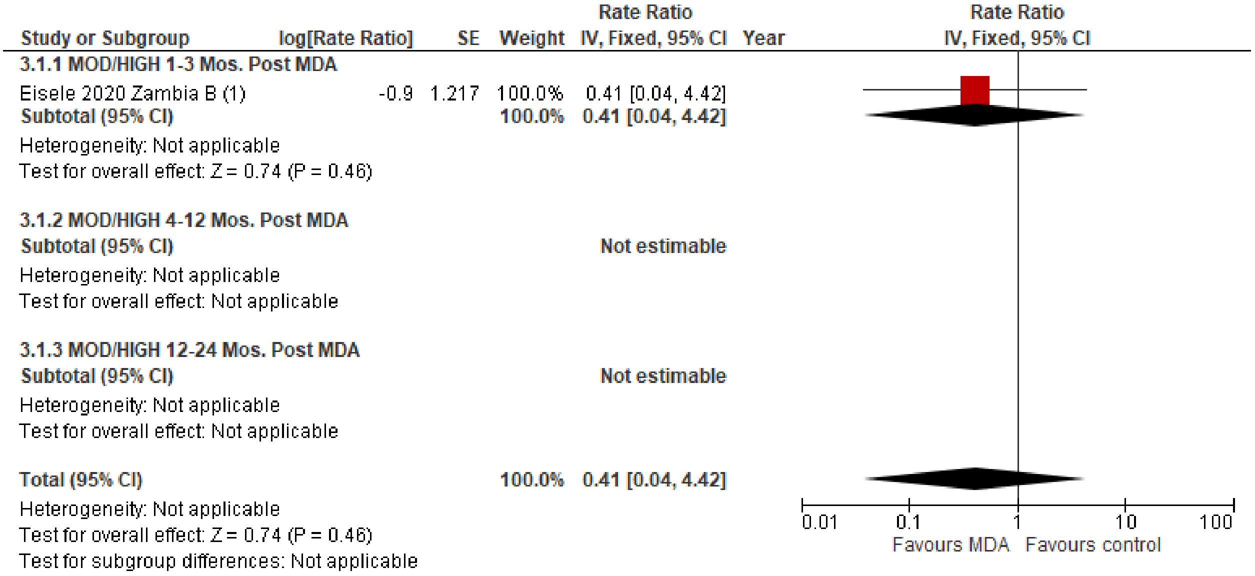

Footnotes
(1) All ages; January-May 2015 and 2016; denominator assumed to be average of 2015 and 2016 mid-year HFCA population

Supplementary figure 4. Incidence of clinical *P. falciparum* malaria in low-very low transmission settings, cRCTs

Study or Subgroup	log[Rate Ratio]	SE	Weight	Rate Ratio IV, Fixed, 95% CI	Year
**4.1.1 LOW/VLOW 1-3 Mos. Post MDA**					
Eisele 2020 Zambia A (1)	-0.684	0.923	11.8%	0.50 [0.08, 3.08]	2014
Morris 2018 Zanzibar (2)	-0.162	1.527	4.3%	0.85 [0.04, 16.96]	2016
**Subtotal (95% CI)**			**16.2%**	**0.58 [0.12, 2.73]**	
Heterogeneity: Chi² = 0.09, df = 1 (P = 0.77); I² = 0%					
Test for overall effect: Z = 0.69 (P = 0.49)					
**4.1.2 LOW/VLOW 4-12 Mos. Post MDA**					
von Seidlein 2019 Myanmar (3)	-0.336	0.474	44.9%	0.71 [0.28, 1.81]	2013
von Seidlein 2019 Cambodia (4)	-3.208	1.01	9.9%	0.04 [0.01, 0.29]	2015
Morris 2018 Zanzibar	-0.079	1.108	8.2%	0.92 [0.11, 8.11]	2016
**Subtotal (95% CI)**			**63.0%**	**0.47 [0.21, 1.03]**	
Heterogeneity: Chi² = 7.05, df = 2 (P = 0.03); I² = 72%					
Test for overall effect: Z = 1.88 (P = 0.06)					
**4.1.3 LOW/VLOW 12-24 Mos. Post MDA**					
Morris 2018 Zanzibar (5)	-0.257	0.697	20.8%	0.77 [0.20, 3.03]	2016
**Subtotal (95% CI)**			**20.8%**	**0.77 [0.20, 3.03]**	
Heterogeneity: Not applicable					
Test for overall effect: Z = 0.37 (P = 0.71)					
**Total (95% CI)**			**100.0%**	**0.54 [0.29, 1.01]**	
Heterogeneity: Chi² = 7.53, df = 5 (P = 0.18); I² = 34%					
Test for overall effect: Z = 1.94 (P = 0.05)					
Test for subgroup differences: Chi² = 0.39, df = 2 (P = 0.82), I² = 0%					

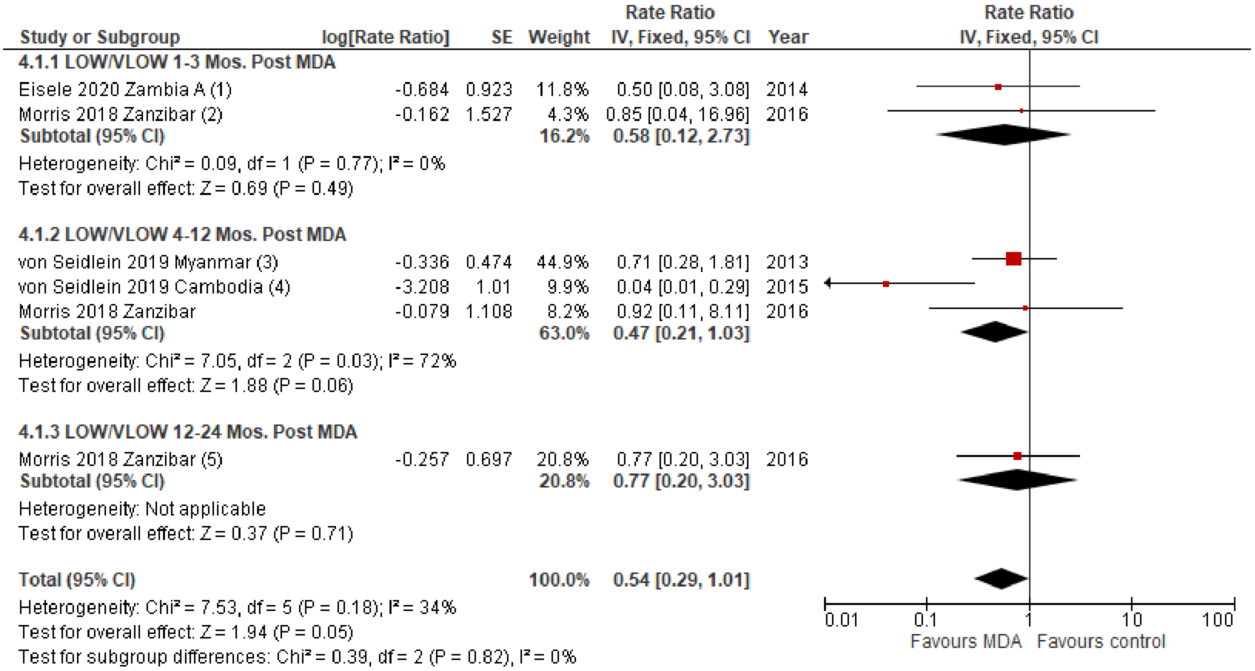

Footnotes
(1) All ages; January-May 2015 and 2016; denominator assumed to be average of 2015 and 2016 mid-year HFCA population
(2) All ages; May-August 2016
(3) All ages; May 2013 to January 2014; *Plasmodium falciparum* or mixed infections
(4) All ages; July 2015 - June 2016; *Plasmodium falciparum* or mixed infections
(5) All ages; May 2016 - August 2017

Supplementary figure 5. Incidence of *P. vivax* malaria, Non-randomized trials

Study or Subgroup	log[Rate Ratio]	SE	Weight	Rate Ratio IV, Fixed, 95% CI	Year
**5.1.1 <1 Months**					
Paik 1974 Solomon Islands B	-1.89	0.12	98.7%	0.15 [0.12, 0.19]	
Gabaldon 1959 Venezuela	-2.83	1.03	1.3%	0.06 [0.01, 0.44]	
**Subtotal (95% CI)**			**100.0%**	**0.15 [0.12, 0.19]**	
Heterogeneity: Chi² = 0.82, df = 1 (P = 0.36); I² = 0%					
Test for overall effect: Z = 15.96 (P < 0.00001)					
**5.1.2 1-3 Months**					
Paik 1974 Solomon Islands B	-0.98	0.08	98.4%	0.38 [0.32, 0.44]	
Gabaldon 1959 Venezuela	-1.73	0.63	1.6%	0.18 [0.05, 0.61]	
**Subtotal (95% CI)**			**100.0%**	**0.37 [0.32, 0.43]**	
Heterogeneity: Chi² = 1.39, df = 1 (P = 0.24); I² = 28%					
Test for overall effect: Z = 12.50 (P < 0.00001)					
**5.1.3 4-12 Months**					
Gabaldon 1959 Venezuela	-1.88	0.41	100.0%	0.15 [0.07, 0.34]	
**Subtotal (95% CI)**			**100.0%**	**0.15 [0.07, 0.34]**	
Heterogeneity: Not applicable					
Test for overall effect: Z = 4.59 (P < 0.00001)					
**5.1.4 12-24 Months**					
**Subtotal (95% CI)**				**Not estimable**	
Heterogeneity: Not applicable					
Test for overall effect: Not applicable					

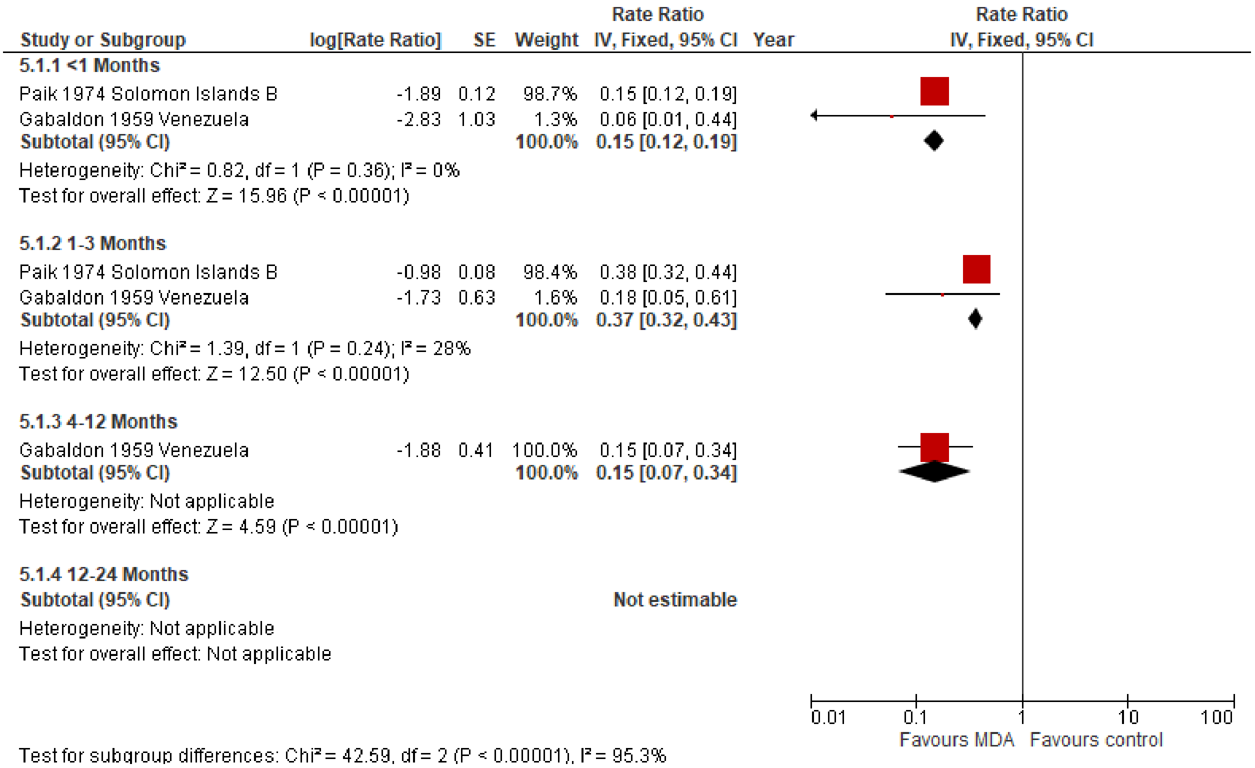

Test for subgroup differences: Chi² = 42.59, df = 2 (P < 0.00001), I² = 95.3%

Supplementary figure 6. Prevalence of *P. vivax* malaria, Non-randomized trials

Study or Subgroup	MDA Events	MDA Total	Control Events	Control Total	Weight	Risk Ratio M-H, Fixed, 95% CI	Year
**6.1.1 <1 Month**							
Paik 1974 Solomon Islands A	8	350	7	99	100.0%	0.32 [0.12, 0.87]	
**Subtotal (95% CI)**		**350**		**99**	**100.0%**	**0.32 [0.12, 0.87]**	
Total events	8		7				
Heterogeneity: Not applicable							
Test for overall effect: Z = 2.24 (P = 0.03)							
**6.1.2 1-3 Months**							
Jones 1958 Kenya (1)	3	135	47	135	79.4%	0.06 [0.02, 0.20]	
Paik 1974 Solomon Islands A	29	655	7	99	20.6%	0.63 [0.28, 1.39]	
**Subtotal (95% CI)**		**790**		**234**	**100.0%**	**0.18 [0.10, 0.33]**	
Total events	32		54				
Heterogeneity: Chi² = 12.57, df = 1 (P = 0.0004); I² = 92%							
Test for overall effect: Z = 5.41 (P < 0.00001)							
**6.1.3 4-12 Months**							
Paik 1974 Solomon Islands A	20	840	7	99	100.0%	0.34 [0.15, 0.78]	
**Subtotal (95% CI)**		**840**		**99**	**100.0%**	**0.34 [0.15, 0.78]**	
Total events	20		7				
Heterogeneity: Not applicable							
Test for overall effect: Z = 2.55 (P = 0.01)							
**6.1.4 12-24 Months**							
**Subtotal (95% CI)**		**0**		**0**		**Not estimable**	
Total events	0		0				
Heterogeneity: Not applicable							
Test for overall effect: Not applicable							

Risk Ratio M-H, Fixed, 95% CI

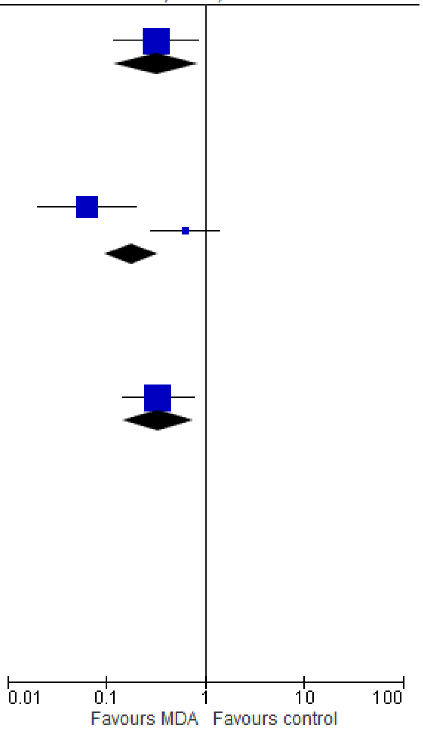

Test for subgroup differences: Chi² = 1.82, df = 2 (P = 0.40), I² = 0%

Footnotes

(1) Jones 1954 KEN: MDA (Pyr every 6 months for 3 rounds) vs. baseline data

Supplementary figure 7. Incidence of clinical *P. vivax* malaria, cRCTs

Study or Subgroup	log[Rate Ratio]	SE	Weight	Rate Ratio IV, Fixed, 95% CI
**7.1.1 4-12 Mos. post MDA**				
von Seidlein 2019 Cambodia (1)	-0.31	0.469	14.6%	0.73 [0.29, 1.84]
von Seidlein 2019 Myanmar (2)	0.426	0.194	85.4%	1.53 [1.05, 2.24]
**Subtotal (95% CI)**			**100.0%**	**1.38 [0.97, 1.95]**
Heterogeneity: Chi² = 2.10, df = 1 (P = 0.15); I² = 52%				
Test for overall effect: Z = 1.78 (P = 0.08)				

Rate Ratio IV, Fixed, 95% CI

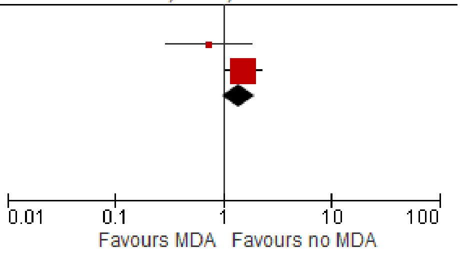

Test for subgroup differences: Not applicable

Footnotes

(1) All ages; July 2015 - June 2016; *Plasmodium vivax*

(2) All ages; May 2013 to January 2014; *Plasmodium vivax*

Supplementary figure 8. Incidence of clinical *P. vivax* malaria, Non-randomized trials

Study or Subgroup	log[Rate Ratio]	SE	Weight	Rate Ratio IV, Fixed, 95% CI
**8.1.2 <1 Mos. Post MDA**				
Cáceres Garcia 2008 Venezuela (1)	-2.04	0.43	1.3%	0.13 [0.06, 0.30]
Simeons 1938 India	-1.47	0.05	98.7%	0.23 [0.21, 0.25]
**Subtotal (95% CI)**			**100.0%**	**0.23 [0.21, 0.25]**
Heterogeneity: $Chi^2 = 1.73$, df = 1 (P = 0.19); $I^2 = 42\%$				
Test for overall effect: Z = 29.75 (P < 0.00001)				
**8.1.3 1-3 Mos. post MDA**				
Cáceres Garcia 2008 Venezuela	0.04	0.21	3.5%	1.04 [0.69, 1.57]
Simeons 1938 India	-1.3	0.04	96.5%	0.27 [0.25, 0.29]
**Subtotal (95% CI)**			**100.0%**	**0.29 [0.26, 0.31]**
Heterogeneity: $Chi^2 = 39.29$, df = 1 (P < 0.00001); $I^2 = 97\%$				
Test for overall effect: Z = 31.89 (P < 0.00001)				
**8.1.4 4-12 Mos. post MDA**				
Simeons 1938 India	-0.33	0.03	100.0%	0.72 [0.68, 0.76]
**Subtotal (95% CI)**			**100.0%**	**0.72 [0.68, 0.76]**
Heterogeneity: Not applicable				
Test for overall effect: Z = 11.00 (P < 0.00001)				
**8.1.5 12-24 Mos. post MDA**				
Simeons 1938 India	-3.2	0.25	100.0%	0.04 [0.02, 0.07]
**Subtotal (95% CI)**			**100.0%**	**0.04 [0.02, 0.07]**
Heterogeneity: Not applicable				
Test for overall effect: Z = 12.80 (P < 0.00001)				

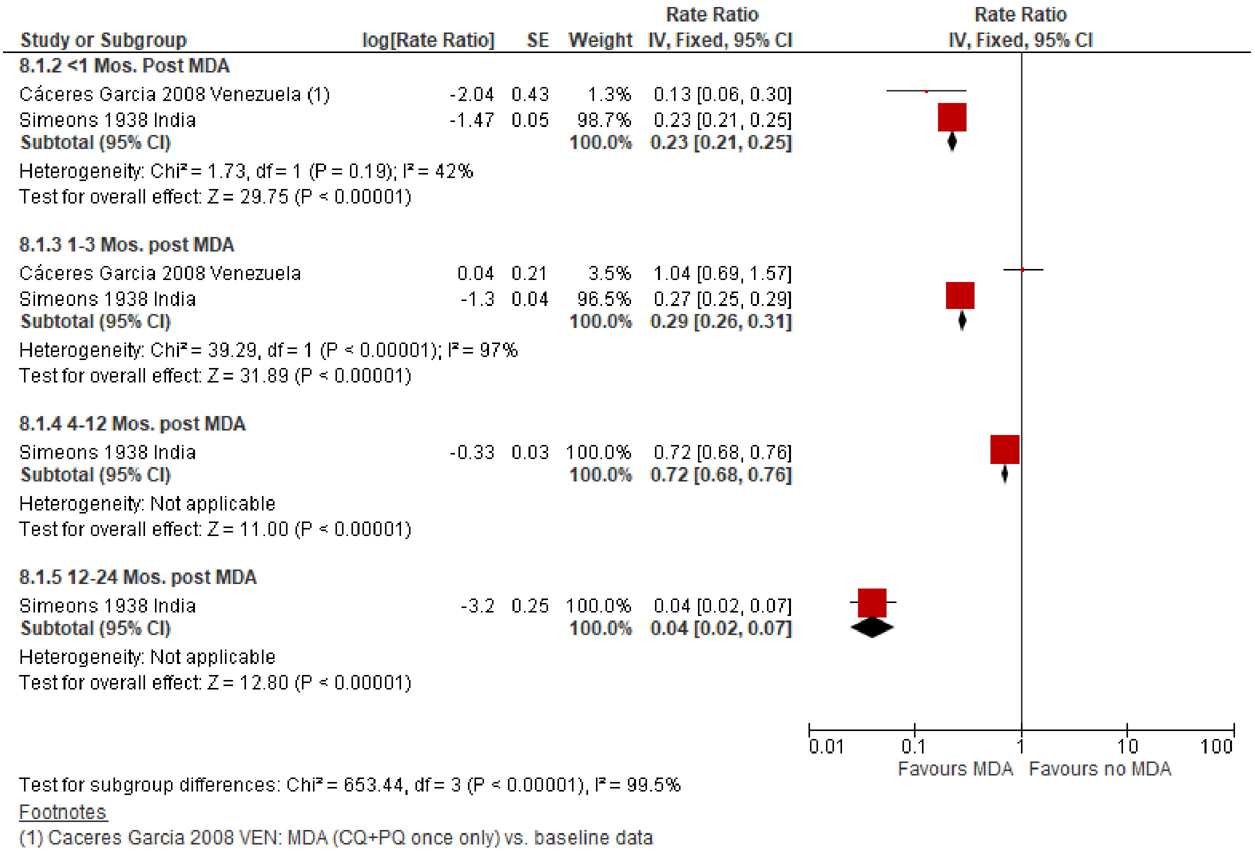

Test for subgroup differences: $Chi^2 = 653.44$, df = 3 (P < 0.00001), $I^2 = 99.5\%$

Footnotes

(1) Caceres Garcia 2008 VEN: MDA (CQ+PQ once only) vs. baseline data

Supplementary figure 9. Serious Adverse Events (Pf and Pv)

Study or Subgroup	Treatment Events	Treatment Total	Control Events	Control Total	Odds Ratio M-H, Fixed, 95% CI
**9.1.1 0-3 Months**					
von Seidlein 2019 \	6	4315	1	2596	3.61 [0.43, 30.03]
**9.1.2 4-12 Months**					
von Seidlein 2019 \	22	4315	9	2596	1.47 [0.68, 3.20]

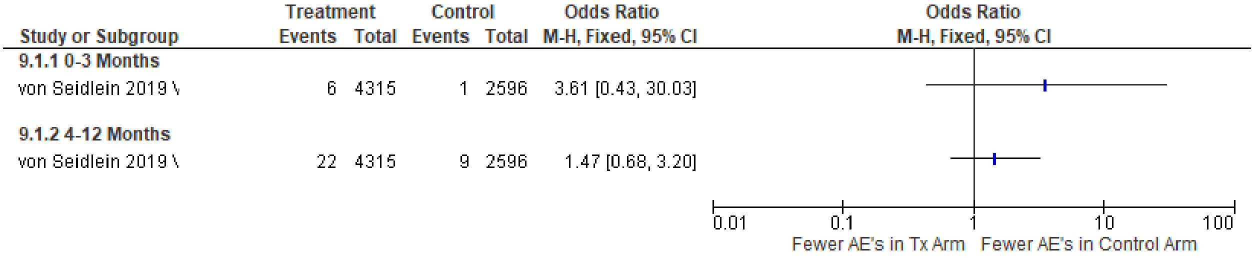

Supplementary figure 10. Proportion of samples with the PfKelch13 mutation among all samples*

Study or Subgroup	MDA Events	MDA Total	Control Events	Control Total	Risk Ratio M-H, Fixed, 95% CI
**10.1.1 Pre-MDA**					
McLean 2021 Myanmar	28	621	27	412	0.69 [0.41, 1.15]
**10.1.2 1-3 Mos. post MDA**					
McLean 2021 Myanmar	6	747	31	485	0.13 [0.05, 0.30]
**10.1.3 4-12 Mos. post MDA**					
McLean 2021 Myanmar	24	1718	25	877	0.49 [0.28, 0.85]
**10.1.4 12-24 Mos. post MDA**					
McLean 2021 Myanmar	33	1992	25	998	0.66 [0.40, 1.11]

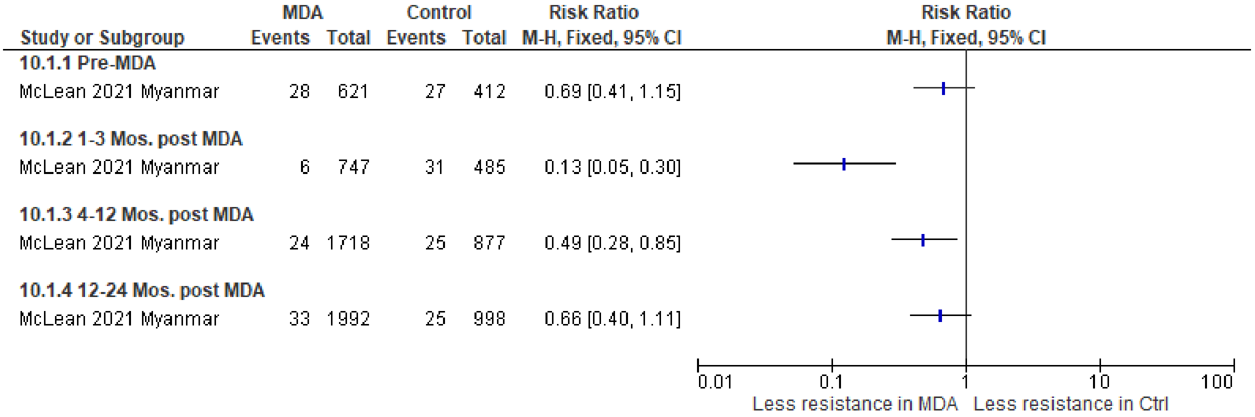

*Data on actual number of samples collected was only available for the pre-MDA and 3 month post MDA time point, for the 4-12 and 12-24 month time points, we used the total number surveyed as the denominator. Numbers from the 5 and 10 month surveys were combined for the 4-12 month period, and numbers from the 15 and 21 month surveys were combined for the 12-24 month period.

Supplementary figure 11. Proportion of samples with the PfKelch13 mutation among Pf positive samples

Study or Subgroup	MDA Events	MDA Total	Control Events	Control Total	Risk Ratio M-H, Fixed, 95% CI
**11.1.1 Pre-MDA**					
McLean 2021 Myanmar	28	52	27	42	0.84 [0.60, 1.17]
**11.1.2 1-3 Mos. post MDA**					
McLean 2021 Myanmar	6	12	31	51	0.82 [0.45, 1.51]
**11.1.3 4-12 Mos. post MDA**					
McLean 2021 Myanmar	24	34	25	41	1.16 [0.83, 1.61]
**11.1.4 12-24 Mos. post MDA**					
McLean 2021 Myanmar	33	43	25	35	1.07 [0.82, 1.40]

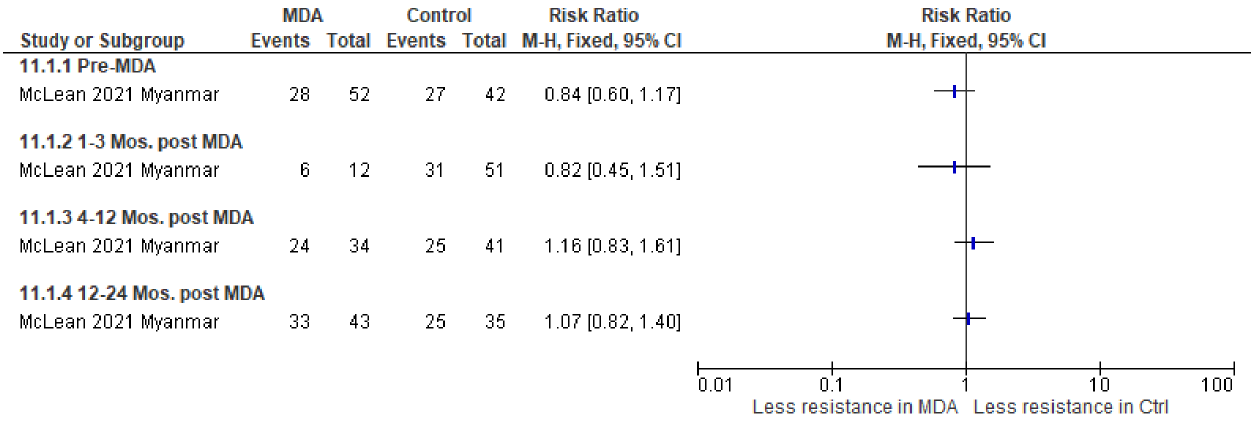

Supplement: Supplemental Materials [file tpmd220766.SD1.pdf]
